# Mental health of the adult population in Germany during the COVID-19 pandemic. Rapid Review

**DOI:** 10.25646/9537.2

**Published:** 2022-02-03

**Authors:** Elvira Mauz, Sophie Eicher, Diana Peitz, Stephan Junker, Heike Hölling, Julia Thom

**Affiliations:** 1 Robert Koch Institute, Berlin Department of Epidemiology and Health Monitoring; 2 Charité-Universitätsmedizin, Institute of Medical Sociology and Rehabilitation Science, Berlin, Germany

**Keywords:** COVID-19 PANDEMIC, SARS-COV-2, MENTAL HEALTH, MENTAL DISORDER, RAPID REVIEW, GENERAL POPULATION

## Abstract

This rapid review examines how the mental health of adults in the general population in Germany changed during the COVID-19 pandemic. We conducted a systematic literature search and included 68 publications as of July 30 2021. The underlying studies were classified according to their suitability for representative statements for the general population and for estimating changes in mental health over time. In addition, the observation period and operationalisation of outcomes were considered.

The first wave of infection and the summer plateau were mapped by 65% of the studies. Studies that were particularly suitable for representative statements due to their research design showed mixed results, which tend to indicate a largely resilient adult population with a proportion of vulnerable individuals. A predominantly negative development of mental health was described by results from more bias-prone study designs. Routine data analyses showed decreases in outpatient and especially inpatient care, increased use of a crisis service, mixed results for outpatient diagnoses, incapacity to work and mortality as well as indications of shifts in the spectrum of diagnoses. As the current evidence is ambiguous, generalised statements should be reflected in favour of a differentiated view. There is a need for research on the further course of the pandemic, specific risk groups and the prevalence of mental disorders.

## 1. Introduction

As a multidimensional stressor, the COVID-19 pandemic and the associated infection control measures pose risks to the mental health of the population at various levels [1–3]. The individual experience of insecurity and anxiety of an infection, the loss of protective factors for mental health (e.g. social and leisure activities, access to care services), the burden of infection protection measures [5–7], the loss of relatives [4] and immediate physical, neurological and psychological symptoms of a COVID-19 infection [8] can have negative consequences for mental health. Longer-term consequences [1, 2] are being discussed in the event of an economic recession caused by the pandemic [9–11]. The increase in risk factors that already existed before the pandemic may also create additional mental health burdens, such as a higher risk of domestic violence [11–13] or increased loneliness [14, 15] in the wake of contact restrictions and widespread closures. In principle, it can be expected that the longer the duration or chronification of stressors, the more difficult it becomes to cope successfully [10].

Against this background, the British Psychiatric Association predicted a ‘tsunami of mental disorders’ as early as May 2020 [17]. Experts also expect an increase in mental stress and disorders in Germany [3, 18, 19]. However, the current findings on the development of the mental health of the population during the COVID-19 pandemic are heterogeneous overall and difficult to assess due to their methodological diversity. Thus, studies from other countries as well as international reviews find, on the one hand, a (partly extreme) increase in psycho-social stress and a subsequent increase in mental disorders [20–26], but on the other hand, no lasting increase in psychopathology [27–30]. In addition to the inconsistency of the results of international studies, conclusions for Germany are also made difficult by significant differences between the countries in the course of the pandemic. In particular, the development of the number of cases and deaths, the resulting burden on the health care system as well as the measures taken to protect against infection, to stabilise the economy and to provide social security differed between the countries.

Since research reacted very quickly to the crisis situation, there is now both a high number of empirical studies and a large variation of the research methodology used [31]. The high demand for reliable findings as a basis for evidence-based policy decisions (e.g. to minimise risks and adapt care services) makes it understandable why rapid solutions are used for immediate data collection or prompt publication of the work on preprint servers even before the quality-assuring peer review. However, this practice makes it necessary to examine the quality and generalisability of the available work for the general population in order to describe the state of research.

This rapid review therefore explores how mental health developed in the adult general population in Germany during the COVID-19 pandemic, taking into account the respective research methodology of the available studies.

## 2. Methododology

Against the background of time and personnel limitations, the methodology of the present review is based on the standards proposed by the Competence Network Public Health on COVID-19 for conducting a rapid review in the context of the current pandemic [32, 33].

### 2.1 Literature search

The search was based on the PECO criteria (Population: General population in Germany; Exposure: COVID-19 pandemic; Comparison: before/after or after COVID outbreak in Germany; Outcome: Mental health) with the following inclusion and exclusion criteria:

#### Inclusion criteria

1. Target population: General population in Germany (and subgroups according to region, age, marital status, employment)
2. Age group: Adult
3. Observation period: During the COVID-19 pandemic
4. Constructs shown: Mental health as the main outcome
5. Publication of a temporal comparison (against measurement time points before the start of the pandemic or during the pandemic)
6. Publication language: German/English

#### Exclusion criteria

1. Publication type: Reviews, Opinions, Comments, Letters to the Editor
2. Methodology: Qualitative data
3. Presentation of study methodology: Methodology not presented with sufficient transparency in published abstracts or on posters
4. Analysis design: Exclusively correlation analyses without reporting of frequencies or their changes in the general population.
5. Target population: Subgroups beyond the above selected sociodemographic characteristics (e.g. people with pre-existing mental disorders, health workers, students).

#### Systematic search

The search was based on the literature database created by the library of the Robert Koch Institute in the course of the COVID-19 pandemic (accessed 30.07.2021). Since the beginning of the pandemic, all publications identified by means of several search strings (Annex Table 1) in the PubMed and Embase databases and the additionally searched preprint servers arXiv, bioRxiv, ChemRxiv, medRxiv, Preprints.org, Research Square and Social Science Research Network (SSRN) have been entered into this database on a weekly basis. The literature database was searched for texts on mental health using two search strings defined by filter terms (Annex Table 1). All texts extracted in this way were filtered for their reference to Germany using a third search string.

#### Offline search

Given the acute need for information with a correspondingly rapid development of scientific publications as well as other dissemination formats, such as reports, study websites, press releases or reports from health insurance funds, it can be assumed that not all findings or publications on the development of mental health in the German general population during the COVID-19 pandemic are already listed in international databases. Based on this, the literature search was extended to include the following areas:

1. Systematic search in the World Health Organization (WHO) database ‘COVID-19. Global literature on corona virus disease’ (Accessed 10.06.2021; Search string see Annex Table 1)
2. Websites of COVID-19 related studies in the general population listed on the website of the German Data Forum (RatSWD) (last update of already identified studies 30.07.2021)
3. Publications and literature lists from the Competence Network Public Health on COVID-19 [35] (last update 30.07.2021)
4. Search via Google search engine (access until 08.07.2021; Search string see Annex Table 1); press releases and current reports or studies by, among others, service providers and suppliers of the health care system (e.g. health insurance funds, Central Institute for Statutory Health Insurance in Germany, hospital statistics).
5. Screening of bibliographies of COVID-19 related reviews, statements and policy briefs for relevant studies and publications (as of 10.06.2021)
6. Screening for relevant COVID-19 related studies and publications listed on the website of the German Society for Psychiatry, Psychotherapy, Neurology and Psychosomatics (DGPPN) (accessed 10.06.2021) [36].

#### Title, abstract and full text screening

After piloting the procedure in the review team and excluding duplicates, the title and abstract screen was carried out by an experienced person (EM). More than 20% of the titles were additionally checked by at least one other person in the team. If the assessment of inclusion or exclusion of a publication was ambiguous, no exclusion was carried out conservatively. All remaining publications were reviewed in full text by at least two people. The publications that were ambiguous in this step were discussed in a team of three people and included or excluded in an iterative process based on the criteria mentioned above. In the course of this process, the first data extraction of the individual publications took place. Information was systematically transferred to a table created for this purpose. For individual publications, the respective data type (primary data, routine data) was identified, on the basis of which the publications were grouped.

### 2.2 Classification of included publications

For a meaningful summary of the findings against the background of the research methodology used and the resulting scope of the empirical results, the included publications were systematised in three ways as follows:

#### 1) Observation periods

In order to assess the extent to which the current state of research allows statements about the entire course of the pandemic since March 2020, the observation periods of the included studies were recorded with the help of half-monthly intervals. Their distribution over the entire period was considered, irrespective of whether results were also reported for them individually. Periods were included for which data were available for at least one week from the respective halves of the month. If this was not the case for any half of the month, the study was assigned to the period in which the most study days occurred. To classify the observation periods, the development of the pandemic in Germany was divided into four phases (wave 1 to 3 and summer plateau 2020) following Schilling et al. [37] as well as Tolksdorf et al. [38] and illustrated with the case and death figures of COVID-19 (Figure 2).

#### 2) Study design of the data used

Two criteria were used to assess the suitability of the study design of the data analysed in the publications for drawing conclusions about the development of mental health in the general population during the COVID-19 pandemic:

#### Criteria 1: Representativeness of the data for the general population

To assess the suitability of the data used in a publication for representative estimates in the general population, primary data was distinguished from routine data.

Routine data are generated in the course of standard documentation and billing in health care processes or in official statistics and represent these as a complete survey without sampling [39]. They are available from different data holders at different levels and thereby differ with regard to their population-based representativeness. For example, data from a single clinic or health insurance fund represent its health care provision without distortion, but may deviate from other clinics or health insurance funds as well as from the nationwide inpatient care or the entirety of persons with statutory health insurance [40].

In primary data collections, a sample of the target population is included in a study. The best approximation to an estimate representative of the general population in Germany is provided by random sampling (probability sampling). Samples can be drawn (a) from the total population with known selection probability [41] or (b) from an access panel with a stratified sampling design if necessary [41, 42]. The persons drawn are invited and motivated to participate. Ideally, measures are implemented to recruit participants from hard-to-reach population groups [41]. Possible biases due to the study design (design effects) and the non-participation of certain population groups (non-response) can be identified and taken into account in the data analysis, for example with weighting factors [43]. In contrast, in studies with non-probability samples (samples without random selection), interested persons participate on their own initiative by accepting non-personalised invitations that are primarily distributed by the media [41]. Thus, participation depends, among other things, on knowing about the study, one’s own motivation to participate and access to the providing medium. These samples can thus be systematically biased by selection effects in a methodologically uncontrollable way and therefore represent less reliable sources of information for the general population [41, 42]. In a methodological study conducted before the pandemic, for example, an overrepresentation of participants with psychological problems by a factor of about 2.5 was found in such a sample [44]. In order to avoid the risk of biased estimates or to be able to assess the uncertainty of the estimates, the use of non-probability samples to study the mental health of the general population during the COVID-19 pandemic is even explicitly discouraged from a methodological point of view [45]. Nevertheless, this pragmatic form of sampling is frequently used and results of corresponding studies find their way into the scientific and public discourse. In order to enable it to be differentiated, the present review differentiated between probability and non-probability samples.

#### Criteria 2: Estimation of changes over time during the pandemic

An essential prerequisite for a robust assessment of change over time is repeated measurement points that are identical in terms of study design (i.e. sample, survey mode, etc.) and outcome measurement. [46]. Trend studies, i.e. repeated and representative cross-sectional or panel surveys with identical study designs, are particularly suitable for this purpose [46, 47]. In these studies, characteristic values collected at different points in time are compared at group level between two samples. In one-off, representative cross-sectional studies, it is possible to assess changes over time by comparing them with norm samples, other reference surveys and retrospective surveys. However, possible sources of error such as recall bias, mode effects or deviating sample composition must be included in the interpretation [48]. Representative cohort or longitudinal studies offer the possibility of identifying changes in specific population groups via intra individual analyses, but can lose representativeness due to the drop out of individuals over time [47, 49].

#### Synopsis of the criteria: Classification in study types

To assess the suitability of the study methodology of the data underlying the publications for assessing temporal changes during the pandemic in the general population, a total of seven study types were defined by combining the two criteria listed above (Annex Table 2):

Within the primary data studies (Category I), the studies with randomly drawn samples were divided into trend studies with random sampling from the general population (study type A) or an access panel (study type B). One-time cross-sectional surveys with random sampling were assigned to study type C, in which the comparison over time took place via retrospective queries or comparisons with other reference surveys without consideration of possible design differences. Cohort studies with random sampling in non-randomly selected regions and without sample augmentation to compensate for drop out were assigned to study type D. Primary data studies with non-probability samples were subdivided into longitudinal studies with repeatedly interviewed participants (study type E) and cross-sectional studies (study type F). Routine data (Category II) were not subdivided, as they are usually available at different points in time and – given the same coding and analysis – can be compared over time.

As comparatively reliable estimates for changes in mental health in the general population during the pandemic, study types A, B, D and, to a limited extent, C, as well as routine data can be contrasted with study types E and F, which are more susceptible to bias.

#### 3) Operationalisation of mental health outcomes

To assess how comprehensive and valid the multi-faceted topic of mental health was captured in the included studies, the mental health outcomes were classified according to the measured construct and its operationalisation. The survey was subdivided into (a) indicators of positive mental health, (b) indicators of mental distress, (c) indicators of acute symptoms of a mental disorder and (d) indicators of health care provision and mortality.

The inventories used to measure the indicators include different possibilities for analysis and interpretation, both with regard to the construct being measured and to pandemic-related changes over time: Non-standardised items or single items of established inventories have limited validity for the measurement of the construct due to a lack of studies on reliability and validity [50]. In contrast, standardised measurement inventories are validated for a defined construct and – given a comparable study design – allow comparison with previous studies and ideally with reference values from norm samples. These also include screening instruments in which currently present symptoms of a mental disorder are queried and thus persons with a high symptom burden can be identified. The frequency of a mental disorder, as it would be diagnosed in a standardized clinical interview, can, however, be both overestimated and underestimated by screening instruments [see e.g. 51]. Documented diagnoses in the health care system (incl. in reports of incapacity for work) presuppose the utilisation of medical or psychotherapeutic services as well as the recognition and documentation of mental disorders by those providing treatment, which means that, among other things, persons with unmet treatment needs are not depicted [52–54].

### 2.3 Systematic extraction of study results

The central results already extracted from the publications in a first step were prepared in tabular form according to the criteria listed here (Annex Table 3, Annex Table 4) and presented in the text. The systematised data extraction was carried out under quality assurance by at least two other independent persons in each case (Annex Table 3).

## 3. Results

### 3.1 Literature search

**Figure 1:**
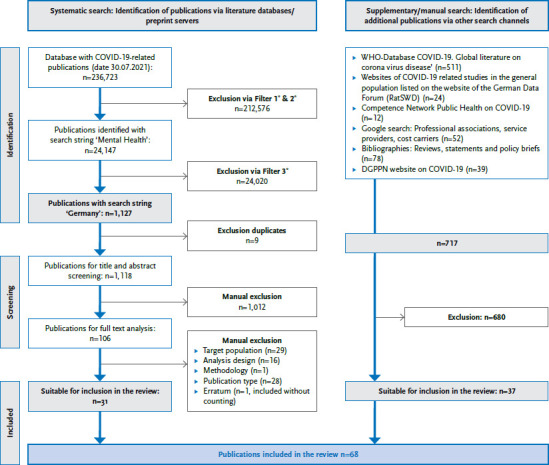
Flowchart on the inclusions and exclusions of the literature search Source: Own table ^*^ Explanation: Filter 1, 2, 3 see Annex Table 1

A total of 1,843 publications relating to Germany were identified using the various search strategies (as of 30.07.2021). With the help of a multi-stage inclusion and exclusion process (Figure 1), 68 publications were included in the review.

### 3.2 Classification of included studies

#### Observation periods

**Figure 2:**
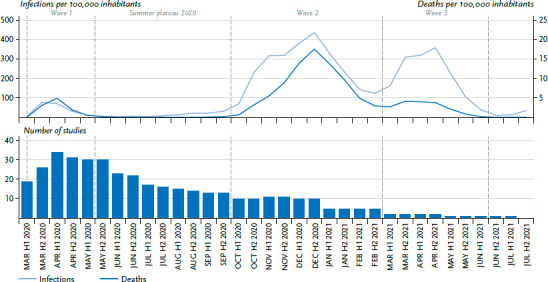
Number of studies included in the review and development of the COVID-19 pandemic in Germany according to incidence and deaths per 100,000 inhabitants Source: COVID-19 cases reported to RKI, own research Source: COVID-19 cases reported to RKI until 2021-08-25, own research, own calculations and graphs. Included are all cases reported to the RKI with age indications ranging from 0 to 120. The division into phases was done based on Schilling et al. [37] and Tolksdorf et al. [38]

At the time of the literature search, the observation periods largely referred to the time of the first wave of infection of the COVID-19 pandemic, which still had relatively low incidence and death rates of people infected with SARS-CoV-2 compared to the later waves (Figure 2). From the second half of March onwards, more than 20 studies were available in each half of the month. For the 2020 summer plateau between the first and second waves, published data from at least 13 studies were also available. However, with the turn of the year 2020/2021, the number of studies decreased sharply: While eight to nine studies were still available in the respective halves of the month for the second wave in 2020, there were only one to five studies for the second and third waves in 2021.

#### Representativeness of the sampling for the general population and the estimation of changes over time during the pandemic

The review included 44 publications based on 25 primary data sources (Category I) and 24 publications based on 18 routine data sources (Category II) (Table 1).

Among the primary data sources, a total of 16 publications from six trend studies with randomly drawn samples from the general population (study type A), five publications from two trend studies with randomly drawn samples from an access panel (study type B) and three further representative cross-sectional studies with one publication each (study type C) were identified. Two publications originated from a cohort study with a random initial sample in non-randomly selected regions (study type D). Four longitudinal studies with five publications (study type E) and nine cross-sectional studies with 13 resulting publications were based on non-probability samples.

In summary, a total of 26 publications based on 12 primary data sources were available in the study types A, B, C and D, which are considered reliable for statements with regard to the general population. In contrast, there were 18 publications from 13 studies in the study types E and F, which are more susceptible to bias. Taking all study categories into account, it became apparent that more than two thirds of all publications (50 of 68) and studies or data sources (30 of 43) could be assigned to the study types (A, B, C, D, routine data) that were assessed as reliable.

### 3.3 Classification and results related to mental health outcomes

#### (a) Indicators of positive mental health

For indicators of positive mental health, results on life satisfaction, well-being and resilience were reported from a total of eight studies in 16 publications (Annex Table 3, Annex Table 4). The measurement was carried out with individual items as well as with standardised inventories. With the exception of study type D, results are available for these indicators from all study types.

For the first pandemic months, stable life satisfaction [16, 62] and stable well-being [16, 56, 65, 67] were reported on the basis of study type A until July 2020 compared to previous years. A later survey showed reduced life satisfaction [63, 64] and a slightly reduced well-being for January and February 2021 [63, 64]. However, the stable median values of the overall group in the first months of the pandemic concealed divergent developments in subgroups: Life satisfaction increased among people with low income or low education, but decreased among the self-employed [66] and people with high education or high income [65] and especially among women [67] as the pandemic progressed [63, 64]. Well-being increased among those living alone, was unchanged among couples without children and single parents, and decreased among couples with children [62].

Similarly, study type B papers reported consistently stable life satisfaction from early March 2020 to mid-July 2021 [74]. At the beginning of the pandemic, resilience was unchanged compared to 2018 values [72, 73], but decreased until early June 2020 [73].

A study of study type C also reported a decrease in life satisfaction in May and June 2020 compared to 2019, which was most evident among women, those with minor children, and those with low education [78].

Study type F papers exclusively reported decreases in life satisfaction in April and May 2020 respectively [97], in well-being in the first weeks of April [95] and for both indicators since October 2020 compared to earlier months in the pandemic [86]. An intra-individual decrease in life satisfaction and positive and negative affect was also observed from March to May 2020 in Study Type E among employed full-time workers [84].

#### (b) Indicators of mental burden

The measurement of feelings of anxiety or dejection, which are counted among the indicators of psychological stress (Annex Table 4), was mainly carried out with individual items from standardised instruments. Results were reported in six publications from three studies. COVID-19 related anxiety and distress were measured with both single-item and standardised instruments and reported in three studies with four publications. Situational distress and psychosocial stress were primarily measured with standardised instruments and reported in a total of 13 publications from eight studies.

Study types A and B consistently showed increased anxiety at the beginning of the pandemic, which decreased in all population groups during April 2020 [61, 72] and subsequently remained stable until March 2021 [72] and July 2020 [59, 60], respectively. In the case of dejection, the slightly increased level at the beginning of the pandemic stagnated until the end of April 2020 [74, 72] and continued to rise until March 2021 in the youngest age group [74]. While no increased psychosocial stress was found in study type A in the Mannheim region for May 2020 [56], it was increased in study type B in March 2020 [74] and changed over time: Anxiety declined until September 2020, increased until the end of April 2021 (especially among young adults) and declined again from May 2021 [74]. Another Type B study showed increased stress levels in February 2021 compared to the summer months of 2020 [70, 71].

Parents of minor children in study type C also reported an increase in stress experience at the time of the highest stress to date compared to January 2020 [76, 77].

Longitudinally, an increase in psychosocial stress was detected in study type D for May 2020 in all age groups. This was stronger in regions with higher incidence and in individuals who tested positive for COVID-19 [80]. Accordingly, in a sample of healthy adults in study type E, intra-individual daily stressors were reduced between the end of March and mid-May 2020 [81, 83]. At the group level, another study of this type found a decrease in COVID-19 specific anxiety from March to June 2020, while longitudinally, an increase was found in about 10% of the sample [82].

Findings from study type F showed elevated psychological distress for almost all reported indicators, which increased over the course of the pandemic. For April to May 2020, increased anxiety [97] and COVID-19 specific fears with a subsequent decrease below the baseline level [93, 94] and a continuous increase [14] for distress were reported. Stress levels were estimated to be moderate [95] in April 2020 and very high [91, 92, 94] in the first months of the pandemic, remaining at a stable high [94] level until July 2020 and increasing steadily until September 2020 [14]. For the first 20 days of the lockdown in April and November 2020, the burden remained high [90].

#### (c) Indicators of acute symptoms of mental disorder

Of a total of 25 primary data studies, 18 used standardised screening instruments to measure acute symptoms of a mental disorder. Results were reported in 31 publications (Annex Table 3 and Annex Table 4). In addition to screening for general psychopathological symptoms, the focus was primarily on depressive and anxiety symptoms.

Either no changes or decreases in psychopathological symptoms were reported from study type A compared to pre-pandemic comparison periods, apart from one finding of higher median scores in depressive and anxiety symptoms in May to June 2020 compared to the same months in 2018 [55]. Nationwide, depressive symptoms remained unchanged in the months after the outbreak compared to the months before [57, 58]. In the Mannheim region, too, no difference was detected in depressive and anxiety symptoms or somatoform disorder symptoms compared to 2018 [56]. A study with two survey periods during the pandemic (April to June 2020 [16, 65, 67] and January to February 2021 [63, 64]) did show an increase in depressive and anxiety symptoms compared to 2019, especially at the level of incident individual symptoms [68]. However, the significantly higher value compared to 2019 was in line with the results from 2016 [16, 65, 67]. With regard to individual symptoms, a decrease in fatigue/lack of energy as well as concentration difficulties was described for the first lockdown in 2020 compared to the same period in 2019 [57, 58]. A decrease in the median value of depressive symptoms was also observed in all population groups during the course of the pandemic from March to July 2020 [59]. Decreases were also described for psychopathological symptoms from mid-March compared to the month before (except for the elderly and persons with low-income) [69].

Results from study type C are based on comparisons with norm samples of the respective inventories and showed no changes in psychopathological symptoms for persons over 65 years of age for April 2020 compared to 2018 [75], while in the group of parents of minor children a slight increase could be found for both depressive and anxiety symptoms (retrospectively assessed for the time of subjectively highest stress) compared to 2010 [76, 77].

In the trend analysis of a longitudinal study of study type D, i.e. when comparing the survey points at group level, the proportions of those with current depressive symptoms or a generalised anxiety disorder were higher in May 2020 than two to seven years earlier in the initial sample [79, 80]. Longitudinally, symptoms increased among persons in the age range 18 to 60 years, but not among the elderly (ibid.). This intra-individual increase was most evident in the youngest age group (20 to 39 years) as well as in regions with a high incidence of infection and among persons tested for COVID-19 (ibid.).

In trend analyses of study type E, reduced psychopathological symptoms were found from the end of March to mid-May 2020 compared to the last measurement point before the pandemic [81, 83], unchanged psychopathology in the first lockdown week in March compared to February 2020 [85] as well as a decrease in depressive symptoms and symptoms of generalised anxiety disorder over the course of April to June 2020 [82]. Despite the findings, the analysis of intra-individual changes consistently pointed to a group of about 8% [83] to 10% [82, 85] of the study participants who experienced an increase in psychological symptoms during the pandemic, and as many as 25% with increases in the severity of depressive symptoms [82]. Another group of 8% to 9% initially developed increased psychological symptoms, which were reduced again within a few weeks [81, 83, 85]. However, the majority reported stable and in some cases even improved mental health (ibid.).

From study type F, elevated levels of psychopathological symptoms were reported almost exclusively. In mid-March to mid-April 2020, the median scores for depressive and anxiety symptoms were significantly elevated in two samples compared to pre-pandemic reference samples and were interpreted as an indication of a possible increase [89] or as an expression of psychological distress [95]. The frequency of depressive symptoms was reported in several studies between 14% and over 35% of participants in the period from March to June 2020 [87, 88, 90–92, 94, 96] and was consistently interpreted as a strong increase in the population [88, 90–92, 94, 96] or as an indication of this [87]. The level was described as stably elevated until the end of July 2020 [94] and a further increase was observed in November 2020 [90]. A comparable strong increase was described at the beginning of the pandemic for symptoms of generalised anxiety disorder with a relative frequency of about 15% to 20% of the participants [88, 90–94, 96]. Immediately afterwards, the symptoms dropped again slightly, but remained at an elevated level in the further course of the pandemic until the end of July [90] and in the second lockdown in November 2020 [94]. The values were interpreted as an up to eight-fold increase compared to 2013 [93] and a two- to ten-fold increase in generalised anxiety disorder compared to reference samples from 2008 and 2017 [94] in the population during the pandemic. In the same period, there were increased sample proportions with symptoms of panic disorder [88, 96] or obsessive-compulsive disorder [96] compared to prevalences from 2012 and 2013 respectively.

#### (d) Indicators of care patterns and mortality

Results on indicators of care provision and mortality were reported in 24 publications from 18 data sources. Various parameters like the use of a crisis service (two publications from one data source), outpatient care for mental disorders (three publications from two data sources), incapacity to work due to mental disorders (nine publications from six data sources), inpatient care for mental disorders (seven publications from six data sources) and mortality in the context of mental disorders (three publications from three data sources) were evaluated.

The utilisation of the crisis helpline ‘TelefonSeelsorge’ at the end of March 2020 showed an increase, which declined again in the following weeks [117, 118]. It particularly concerned counselling topics in the spectrum of health, relationships and violence and was higher in federal states with stricter infection control measures [118].

In outpatient mental health care, an increase in GPs’ first diagnoses of anxiety disorders was reported in the period March to June 2020, affecting more people aged over 30 years and with diagnosis of asthma and COPD [106]. In contrast, a decrease was reported for outpatient first diagnoses of depression among persons aged ≥65 years, for whom physician contacts, referrals and hospital admissions from psychiatric or neurological practices also declined [107]. There were fluctuations in the number of treatment cases among medical and psychological psychotherapists and specialists in psychosomatic medicine and psychotherapy. This fell below the previous year’s level from mid-March to the end of May and from November to the end of 2020 [121]. In June 2020, only these two groups of doctors showed a significant increase. Declines were also recorded in psychotherapeutic individual and group therapies as well as substitution therapy for drug addiction, which remained consistently below the 2019 level from mid-March 2020 [121].

Developments in incapacity to work due to mental disorders (IW) differed between the health insurance funds. BARMER [101] and BKK[103] observed a decrease in sick leave in the first months of the pandemic. DAK [104] and AOK [99] found a decrease in incapacity to work cases in 2020. In contrast, the KKH registered an increase in the number of cases of incapacity for work (IW) in the first half of 2020 [108] and the TK in the number of days of IW in 2020 [119, 120]. The BKK also recorded an increase in the number of sick days in November 2020 and at the beginning of 2021 [103]. According to the KKH, the case duration increased overall in 2020 [109, 110], whereby according to the DAK, short incapacity for work cases decreased in 2020, while cases with a duration of over six weeks increased [104]. The DAK reported shifts in the spectrum of diagnoses causing incapacity for work in 2020, with increases in anxiety disorders, reactions to severe stress and adjustment disorders [104].

In the area of inpatient care for mental disorders, there were decreases in the scope and shifts in clinical characteristics: For AOK-insured persons, the number of inpatient cases in psychiatric, psychotherapeutic and psychosomatic clinics and departments fell below the 2019 level from March 2020 to February 2021 [100]. From mid-March to early April 2020, the decline in diagnoses of mental and behavioral disorders (as coded in ICD-10 chapter V) was rather high compared to other indications [98]. One hospital group also reported a decline in admissions to day clinic and inpatient admissions from mid-March to the end of May 2020 [102], which affected various main diagnosis groups to varying degrees. At the same time, the length of stay of inpatient cases with coded diagnoses of mental and behavioral disorders fell considerably in another hospital network [111]. For psychiatric emergencies, there was a decrease in presentations [111, 113, 114] in the period from the beginning of the pandemic to the end of May, and only one hospital found no change in the absolute number of psychiatric emergencies [112]. At the same time, an increase in repeat presentations for psychiatric emergencies and changes in diagnostic spectrum and psychopathological findings were observed, with formal thought disorder, hopelessness and social withdrawal documented more frequently, while suicidality remained unchanged [114]. Among psychiatric emergencies [114] and psychiatric consultations [112] where a substantive relationship of the complaints to the COVID-19 pandemic was identified, the proportion of persons with suicide attempts was increased compared to cases unrelated to the COVID-19 pandemic.

A change in mortality in the context of mental disorders is evident for the number of deaths due to intoxication, which was higher in 2020 than in 2019 nationwide [105]. In contrast, for suicide rates in the city of Leipzig, no differences can be detected between different phases of infection control measures (light vs. heavy restrictions) nor to the temporal developments of previous years [116]. In preliminary evaluations, the nationwide suicide rates show a slight decrease in cases for 2020 compared to 2019 [115].

## 4. Discussion

Given the extensive and diverse body of studies, this rapid review aims to provide a comprehensive assessment of the development of the mental health of the adult population in Germany during the COVID-19 pandemic. For this purpose, as of July 30 2021, 68 relevant publications were identified and included in the review. The data used in the publications was classified with regard to the observation period, the research methodology used to answer the research question and the content. Based on the extracted main results, the state of research is summarised in the following and further research needs are derived.

The data of 65% of the included publications referred to the first wave of infection and the summer plateau 2020. In comparison, the level of information for the second and third waves from autumn 2020 to summer 2021 is much more limited and limits a comprehensive assessment of the entire course of the pandemic.

At the present time, the primary data studies show a large variation of the research methodology used across the study types A to F as defined here. More than half of the studies conducted and slightly less than half of the publications resulting from them were accounted for by study types E and F. These do not represent reliable sources of information for the general population due to the potential bias of the results, even though they can provide valuable information through the analysis of correlations or, above all, intra-individual changes. Despite their high visibility in scientific discourse due to a relatively high number of publications, these results should be placed into context with current studies less prone to bias and thus be interpreted with caution. Compared to primary data analyses, routine data analyses also evaluated a high number of data sources, from which, however, far fewer publications emerged.

From the broad spectrum of mental health topics, the studies conducted primarily focused on indicators of a current symptoms of a mental disorder using validated screening inventories. Indicators of mental distress and positive mental health ranked second and third, respectively. No study used standardized diagnostic procedures to determine the frequency of mental disorders according to established classification systems. Consequently, no evidence-based statement can yet be made, about the development of the prevalence of mental disorders in particular.

The comparison of results across all indicators indicates dependencies on the study design: The cross-sectional study types A, B and C, which were assessed as more suitable for a reliable estimation for the general population due to a representatively designed random sample, reported predominantly mixed results. If anything, the results suggest that the adult general population was rather resilient during the study period and that their mental health remained relatively stable despite increasing stress. First included publications with more recent data collection pointed to a deterioration in life satisfaction from the turn of the year 2020/2021. The necessity of a socially differentiated approach was illustrated by findings showing that a stable population mean value is based on diametrically opposed developments in different population groups. From the more bias-prone study type F with non-probability samples, predominantly results of a negative development of mental health and a strong increase of stress were reported. However, against the background of possible selection effects [42, 44, 45] compared to the general population, these findings should be interpreted cautiously as indications of changes in individual subgroups that need to be identified more precisely. In longitudinal studies of type D and E, both intra-individual deterioration and improvement of mental health were observed. Again, longitudinal analyses indicated that stable scores over time in the overall group may be due to contrasting trends in subgroups. Results based on routine data analyses in the context of mental health showed predominantly decreases in outpatient and inpatient case numbers or services, increases in the use of a crisis service, mixed results for the development of outpatient diagnoses, incapacity to work and mortality, as well as indications of shifts in the diagnostic spectrum and in clinical characteristics of treated cases.

In summary, the current scientific evidence can be considered inconclusive. Taking into account the study methodology used, statements of a dramatic deterioration in the mental health of the adult population during the COVID-19 pandemic in Germany in particular should therefore be questioned in favour of a more differentiated view.

Comparable to this, the first international systematic reviews also reported a heterogeneous picture [31]. In addition to significant increases [24] and decreases [27] in psychopathological symptoms, there was a tendency for mental health problems to increase at the beginning of the pandemic, which decreased [30] almost to the initial level after a few weeks. This observation corresponds to the findings from study types A, B and C outlined for Germany. Results of opposing trends in subgroups analogous to the findings from study types A and E were also reported [122]. However, a comprehensive evaluation of the international findings, in consideration of research methodology, regional differences in the course of the pandemic and special features prior to the outbreak, is still pending.

A psychological reaction of people to a crisis as profound as the global COVID-19 pandemic is to be expected within an appropriate and healthy range of experience and behaviour. Reduced well-being, increased mental distress or partly transient (single) symptoms of mental disorders alone do not imply a need for clinical treatment compared to manifest mental disorders with long-term functional limitations [31]. Since the development of mental disorders requiring treatment is often preceded by chronic overload and stress with a longer incubation period, measures should nevertheless be taken to prevent them and to promote mental health [see e.g. 3, 123], as implied by the available research on the experience of stress.

The limitations of the present rapid review include the following aspects: As the focus was on the mental health of the general population, studies in specific populations such as students, people with mental disorders or health workers were not included. Not all sources of potential bias were considered in the classification of included studies (e.g. survey mode, response or drop-out rates [41]). Furthermore, no established risk of bias tool was used, as systematic biases were explicitly considered as an object of the review question.

In principle, it should be noted that trends that already existed before the pandemic were not distinguished from temporal changes during the pandemic in most publications, neither empirically nor in the discussion of results. The latter can therefore not always be interpreted as causally linked to the pandemic.

From the present results, focal points of the current need for research can be derived:

1. For a comprehensive assessment of the course of the pandemic so far, results are needed for the second and third waves of infection. Since the increase in the number of cases and deaths as well as the (continued) duration of infection protection measures were comparatively higher during the latter, changed and increased risks for mental health can be assumed compared to the first wave, such as the chronification of stressors as well as the continued loss of resources [3]. Surveillance of the mental health of the population is also necessary after the infection waves have subsided, among other things because mental disorders can be preceded by longer sub-clinical or prodromal phases (precursor phases) and thus there may be a time-delayed increase in psychopathology [3]. In addition, it should be noted that in the past, economic recessions have been associated with an increase in mental disorders and suicides in the population, which may also contribute to an increased burden of disease in the context of a pandemic [2, 9, 10, 124].
2. Similar vulnerable and highly stressed groups for the development or worsening of psychopathological symptoms are described across all study designs and also across countries. In addition to people with mental disorders and health workers, these include young people, women and families with young children. International reviews also point to a widening of social inequalities in mental health in the wake of the pandemic by education, insecure income or unemployment [125]. The systematic identification of risk groups in Germany could take place in a second step on the basis of the present research. It is necessary for target group-oriented health promotion, prevention and care planning and could be included in future pandemic and crisis management.
3. Internationally, too, it is demanded that studies whose design allows a reliable and differentiated assessment of temporal changes in the general population should be carried out as a matter of priority [45, 126]. Trend and cohort studies in particular can contribute to the identification of subgroups that are particularly affected or at risk and test previous indications of the reversibility of negative effects as well as favourable factors. The results should be reported in a socially stratified manner in order to uncover possibly opposing developments in different population groups.
4. Research gaps remain for a variety of aspects of mental health, including key indicators such as incidence and prevalence of mental disorders and functional limitations, burden of disease and mortality associated with mental distress and disorders. While the standardised diagnosis of mental disorders before the pandemic showed a stable prevalence in the population (survey 1997–1999 vs. 2009–2012 [127, 128]), the assessment of current changes in care needs requires a renewed psycho-diagnostic data collection.
5. Finally, the direct effects of contracting COVID-19 or requiring intensive medical treatment [129, 130] may also have a negative impact on mental health and may be reflected in changes at the population level in the short or long term, especially against the background of higher case numbers during the later waves of infection. Consequently, population-based studies should capture these two events where possible and take them into account when examining mental health trends.

In the coming months, the publication of further research results on mental health in Germany during the COVID-19 pandemic can be expected. Since methodologically high-quality studies in particular require a longer planning phase, the longer the pandemic lasts, the greater the number of studies that allow valid statements at the population level as well as over time and that contribute to closing some of the research gaps mentioned. For effective mental health surveillance, study results must be systematically pooled and comparatively evaluated on an ongoing basis. Knowledge of the current situation and the temporal development dynamics of mental health in Germany can contribute to actors from health promotion, prevention, care and relevant political departments developing targeted and evidence-based approaches in order to manage the COVID-19 pandemic.

**Corrigendum, page 2**

For the first author, the following affiliation was added: Charité-Universitätsmedizin, Institute of Medical Sociology and Rehabilitation Science, Berlin, Germany.

## Key statements

- A variety of studies with divergent research methodologies examined the development of mental health during the COVID-19 pandemic.
- Study results differed depending on the study design used, among other factors.
- Despite increasing stress, the results tend to show relatively stable mental health in the adult population.
- Stable values over time in the overall group are partly due to opposing trends in subgroups.
- Systematic mental health surveillance is needed for evidence-based crisis management during and after the COVID-19 pandemic.

## Figures and Tables

**Table 1:** Included studies and publications by category and study type Source: Own table

Category	Study type	Included publications		
		Number of studies or data sources	Number of publications	References
**Total**		**43**	**68 plus Erratum**	**[14, 16, 55–121]**
**I Primary data analysis**				
**Total**		**25**	**44**	**[14, 16, 55–97]**
A		6	16	[16, 55–69]
B		2	5	[70–74]
C		3	3 plus Erratum	[75–78]
D		1	2	[79, 80]
E		4	5	[81–85]
F		9	13	[14, 86–97]
**II Routine data analysis**				
**Total**		**18**	**24**	**[98–121]**

## Appendix

**Annex Table 1:** Applied search strings in databases Source: Own table

DATA BANK	SEARCH STRATEGY
Weekly updated database of studies related to the COVID-19 pandemic (as of 30.07.2021)	**Search string PUBMed 1:** (‘Severe Acute Respiratory Syndrome Coronavirus 2’ [Supplementary Concept] OR ‘COVID-19’ [Supplementary Concept] OR ‘covid 19 diagnostic testing’ [Supplementary Concept] OR ‘covid 19 drug treatment’ [Supplementary Concept] OR ‘covid 19 serotherapy’[Supplementary Concept] OR ‘covid 19 vaccine’ [Supplementary Concept] OR ‘Severe Acute Respiratory Syndrome Coronavirus 2’[tiab] OR ncov*[tiab] OR covid*[tiab] OR sars-cov-2[tiab] OR ‘sars cov 2’[tiab] OR ‘SARS Coronavirus 2’[tiab] OR ‘Severe Acute Respiratory Syndrome CoV 2’[tiab] OR ‘Wuhan coronavirus’[tiab] OR ‘Wuhan seafood market pneumonia virus’[tiab] OR ‘SARS2’[tiab] OR ‘2019-nCoV’[tiab] OR ‘hcov-19’[tiab] OR ‘novel 2019 coronavirus’[tiab] OR ‘2019 novel coronavirus*’[tiab] OR ‘novel coronavirus 2019*’ [tiab] OR ‘2019 novel human coronavirus*’[tiab] OR ‘human coronavirus 2019’[tiab] OR ‘coronavirus disease-19’ [tiab] OR ‘corona virus disease-19’[tiab] OR ‘coronavirus disease 2019’[tiab] OR ‘corona virus disease 2019’[tiab] OR ‘2019 coronavirus disease’[tiab] OR ‘2019 corona virus disease’[tiab] OR ‘novel coronavirus disease 2019’ [tiab] OR ‘novel coronavirus infection 2019’[tiab] OR ‘new coronavirus*’[tiab] OR ‘coronavirus outbreak’[tiab] OR ‘coronavirus epidemic’[tiab] OR ‘coronavirus pandemic’[tiab] OR ‘pandemic of coronavirus’[tiab]) AND (‘2019/12/01’[PDAT] : ‘2099/12/31’[PDAT])
	**Search string PUBMed 2:** (‘wuhan’[tiab] or china[tiab] or hubei[tiab]) AND (‘Severe Acute Respiratory Syndrome Coronavirus 2’[Supplementary Concept] OR ‘COVID-19’ [Supplementary Concept] OR ‘covid 19 diagnostic testing’[Supplementary Concept] OR ‘covid 19 drug treatment’[Supplementary Concept] OR ‘covid 19 serotherapy’[Supplementary Concept] OR ‘covid 19 vaccine’[Supplementary Concept] OR ‘coronavirus*’[tiab] OR ‘corona virus*’[tiab] OR ncov[tiab] OR covid* [tiab] OR sars*[tiab])
	**Search string Embase 1:** (‘severe acute respiratory syndrome coronavirus 2’:ti,ab OR ‘severe acute respiratory syndrome coronavirus 2’/exp OR ‘covid 19’/exp OR ncov*:ti,ab OR covid*:ti,ab OR ‘sars cov 2’:ti,ab OR ‘sars-cov-2’:ti,ab OR ‘sars coronavirus 2’:ti,ab OR ‘sars coronavirus 2’/exp OR ‘severe acute respiratory syndrome cov 2’:ti,ab OR ‘wuhan coronavirus’: ti,ab OR ‘wuhan seafood market pneumonia virus’:ti,ab OR sars2:ti,ab OR ‘2019-ncov’:ti,ab OR ‘hcov-19’:ti,ab OR ‘novel 2019 coronavirus’:ti,ab OR ‘2019 novel coronavirus*’:ti,ab OR ‘novel coronavirus 2019’/exp OR ‘2019 novel human coronavirus*’:ti,ab OR ‘human coronavirus 2019’:ti,ab OR ‘coronavirus disease-19’:ti,ab OR ‘corona virus disease-19’:ti,ab OR ‘coronavirus disease 2019’:ti,ab OR ‘coronavirus disease 2019’/exp OR ‘corona virus disease 2019’:ti,ab OR ‘2019 coronavirus disease’:ti,ab OR ‘novel coronavirus 2019*’:ti,ab OR ‘novel coronavirus disease 2019’:ti,ab OR ‘novel coronavirus infection 2019’:ti,ab OR ‘2019 corona virus disease’:ti,ab OR ‘new coronavirus*’:ti,ab OR ‘coronavirus outbreak’:ti,ab OR ‘coronavirus epidemic’:ti,ab OR ‘coronavirus pandemic’:ti,ab OR ‘pandemic of coronavirus’:ti,ab OR ‘severe acute respiratory syndrome coronavirus 2 vaccine’/exp OR ‘covid 19 vaccine’/exp) AND (2020:py OR 2021:py)
	**Search string Embase 2:** (wuhan:ti,ab OR china:ti,ab OR hubei:ti,ab) AND (‘severe acute respiratory syndrome coronavirus 2’:ti,ab OR ‘severe acute respiratory syndrome coronavirus 2’/exp OR ‘severe acute respiratory syndrome coronavirus 2’ OR ‘covid*’:ti,ab OR ‘covid 19’/exp OR ‘covid 19’ OR coronavirus*:ti,ab OR ‘corona virus*’:ti,ab OR ncov:ti,ab OR covid*:ti,ab OR sars*:ti,ab OR ‘sars coronavirus 2’/exp) **+ additional hand research/evaluation of the preprint servers arXiv, bioRxiv, ChemRxiv, medRxiv, Preprints.org, Research Square and Social Science Research Network (SSRN)**
Endnote Smartgroups	Search string ‘Mental Health’: Smartgroup 1: Mental OR psychological OR psychosocial OR Psychisch OR psychische OR psychischer OR psychischen OR depression OR anxiety OR Angst Smartgroup 2: mentale OR Resilienz OR resilience OR Lebenszufriedenheit OR life satisfaction OR PTSD OR Wellbeing OR Wohlbefinden OR Suizid OR suicide Search string ‘Deutschland’: Smartgroup 3: German OR Germany OR deutsch OR Deutschland OR deutsche OR deutscher
Data bank of the World Health Organization (08.06.2021) https://search.bvsalud.org/global-literature-on-novel-coronavirus-2019-ncov/	Search string as a combination of the endnote search terms (tw:(Mental OR psychological OR psychosocial OR Psychisch OR psychische OR psychischer OR psychischen OR depression OR anxiety OR Angst OR mentale OR Resilienz OR resilience Lebenszufriedenheit OR life satisfaction OR PTSD OR Wellbeing OR Wohlbefinden OR Suizid OR suicide)) AND (tw:(German OR Germany OR deutsch OR Deutschland OR deutsche OR deutscher))
Google search	ENGLISH: (Covid OR Corona OR pandem*) AND German* AND (Mental OR disorder OR wellbeing OR distress OR substance OR missuse OR violence OR abuse) GERMAN: Covid AND German* AND Psych* Krankenkasse OR Krankenversicherung OR Routinedaten AND Corona OR Covid AND Psych

**Annex Table 2:** Classification of study type Source: Own table

		Random sample of the general population	Random sample from an access panel	Adaptation and design weighting	Non-Probability Sample	Trend Design	Cohort Design	Identical assessment of the outcome over several measurement points
Study type I: PRIMARY DATA								
**A**	Representative cross-sectional study with random sample from the general population and possibility of comparison with identically surveyed cross-sectional study in trend	X		X		X	In part	X
**B**	Representative cross-sectional study with random sample from an ACCESS-Panel and possibility of comparison with identically surveyed cross-sectional study in trend		X	X		X	In part	X
**C**	Representative cross-sectional study with random sample and one-time survey	X						
**D**	Cohort study with random sample at t1	X					X	X
**E**	Cohort study with non-probability sample at t1				X		X	X
**F**	Cohort study with non-probability sample				X			In part
**Study type II: ROUTINE DATA**								

**Annex Table 3 Publication:** Category I: Primary data Abbreviations used: M = median, SD = standard deviation, cut-off = fixed limit of an instrument from which a certain interpretation of the results applies, n = sample size, N = full survey, OP = observation period, CP = comparison period, t1 = measurement time 1, t2 = measurement time 2., difference = calculation of the intra-individual deviation of different measurement times

STUDY TYPE A: Representative cross-sectional studies in trend design with random sampling from the general population and identical survey design of the temporal comparative data [1–16]				
Study or data basis and publications (including observation period etc.)	Results			
	Outcome		Operationalisation/measuring instrument	Description and interpretation by authors of the publication
**Data basis: SOEP-CoV** Survey study (telephone): special study with two survey waves of the Socio-Economic Panel (SOEP) based on offline random sampling (households of the general population, persons aged 18-over 80); comparison over time between the samples and additionally with identically surveyed outcomes in previous years’ SOEP surveys. First wave: 31.03.–04.07.2020; second wave: 01.01.–28.02.2021 [1] Entringer T, Krieger M (2020): OP: 01.04.–30.06.2020 (n=5,529, division into three pandemic phases) [2] Entringer T, Kröger H (2020): OP: 01.04.– 26.4.2020 (n=3,599) [3] Entringer T, Kröger H, Schupp J et al. (2020): OP: 01.04.–26.04.2020 (n=3,599) [4] Kritikos AS, Graeber D, Seebauer J (2020): OP: 01.04.–24.05.2020 (n=3,052) [5] Liebig S, Buchinger L, Entringer T et al. (2020): OP: 01.04.–04./05.07.2020 (n=5,617) [6] Seebauer J, Kritikos AS, Graeber D (2021): OP: 01.04.–04./05.07.2020 versus CP 2019 (n=5,617) [14] Entringer T, Kröger H (2021): OP: 01.01.–28.02.2021 versus CP: 31.03.–04.07.2020 or previous years (n=6,013) [13] Entringer T, Kröger H (2021): OP: 01.01.–28.02.2021 versus CP: 31.03.2020–04.07.2020 or previous years (n=6,013)	a	Satisfaction of life compared to 2015–2019	Short scale L-1 (Scale 0–10)	**Unchanged** in general population until 30.06.2020 at 7.44 similar to previous year [2]; **reduction** at the beginning of 2021 to 7.2 [13, 14] **Reduction** among self-employed [4]; especially **reduction** among women [5] also in further course of the pandemic [13, 14] **Increase** for single people; **unchanged** among couples without children and single parents; **reduction** for couples with children [1] **Increase** among people with low income, low education; **decrease** among people with high income, high education – reduction of socioeconomic differences [3], is also evident as the pandemic continues [13]
	a	Wellbeing compared to 2007–2019	Four single items (Scale 1–5)	**Unchanged** in general population [2, 3, 5]: 14.7 in 2020, similar to previous year. Small and not significant **reduction** to 14.5 in 2021 [13, 14] Increase for single people; **unchanged** among couples without children and single parents; **reduction** for couples with children [1]
	c	Depressive and anxiety symptoms compared to 2016, 2019	PHQ-4 (M, Scale 1–12)	**Increase** in general population compared to 2019; however, **unchanged** compared to 2016 [2, 3, 5]. In the OP 01.04–30.06.2020, the value is 2.4 [2, 3, 5] and in the OP 01.01–28.02.2021, the value is 2.2 [13, 14]. In 2019 it was 1.9 and in 2016 it was 2.3 **Increase** of incidence of depressive symptoms in general population compared to 2019: 21% depressive symptoms not present at any time in OP vs. 38% in CP [6] Particularly affected by an **increase** are people with a migration background [3, 13, 14], women and younger people [13, 14]; longitudinally, the situation worsened considerably for female self-employed workers [6]
**Data basis: Mannheim Corona Study** Survey study (online): special study of the German Internet Panel (GIP) based on offline random sampling: repeated weekly survey of approx. 3,600 people aged 18–75 in each case in the period 20.03.–09.07.2020, daily survey of an average of 489 people; comparison over time by showing weekly progression [7] Mata J, Wenz A, Rettig T et al. (2020): OP: 20.03.–09.07.2020 [8] Blom A G, Wenz A, Rettig T et al. (2020): OP: 20.03.–09.07.2020 [9] Naumann E, Mata J, Reifenscheid M et al. (2020): OP: 20.03.–16.04.2020	b	Anxiety	State-Trait Anxiety Inventory short scale Until week 4: Five items (M; cut-off >2) [9] from week 5: Two items [7, 8]	**Decrease** of the initially increased level of anxiety in the general population already during the first lockdown from 20 March to 16 April to 1.9 [9], in the further course of the pandemic until July stable, very slightly decreasing level to 1.70 and 1.66 respectively (taken from graphical representation) [7, 8] **Decline** in all population groups, strongest among young people [9]
	c	Depressive symptoms	from week 5: PHQ-2	**Decrease** in general population over the course of the pandemic [7] from 1.50 to 1.43 (from graphical representation) in all population groups, no increase in vulnerable groups
**Data basis: GEDA-EHIS 2019/2020** Survey study (telephone) of the European Health Interview Survey (EHIS) for Germany in the general population aged 15-over 80 (n=23,001, approx. 1,300 persons per month); comparison over the time before, during and after the lockdown in Germany (from 16.03.2020) within the surveyed study sample and presentation of monthly progression [11] Damerow S, Rommel A, Prütz F et al. (2020): OP: 04/2010–09/2020 (n=23,001) versus CP: 04/2019–03/2020 [15] Cohrdes C, Yenikent S, Wu J et al. (2021): OP: 01.01.–30.06.2020 versus CP: CW 1–11 2020, CW 12–18 2020, CW 19–31 2020 (n=9,011)	c	Depressive symptoms	PHQ-8 (>9)	**Unchanged** in general population [11] 6.6% of population in CW 15–26 2020 compared to 8.3% CW 15–26 2019.
	c		Individual symptoms PHQ-8 over time	**Decrease** of two symptoms in general population (lack of energy; concentration problems) [11] 18.1% of population report concentration problems (CW 15–26 vs. 21.9% CW 15–26 2019) [11] and 50.7% of population report lack of energy (CW 15–26 vs. 64.0% CW 15–26 2019) [11] **Decrease** in lack of energy in general population during CW 12–18 2020 (first lockdown) compared to CW 1–11 2020, subsequent **increase** in CW 19–31 2020 to level of CW 1–11 2020 [15]
**Data basis: Study of Ulm University** Survey study (personal interview) using the random route method in the general population aged 14–95; temporal comparison of the sample surveyed before and after the lockdown in Germany (from 16.03.2020) [12] Sachser C, Olaru G, Pfeiffer E et al. (2021): OP: 10.02.–15.03.2020 versus CP 16.03.–25.04.2020 (n=2,503)	c	Psychopathological symptoms	Brief Symptom Inventory (BSI-18)	**Decrease** in general population from M=3.73; SD=6.39 compared to M=5.00; SD=7.93 in the CP [12] **Unchanged** in OP compared to CP for older people and people with low income
**Data basis: Study of the Universities of Mainz and Leipzig** Survey study (personal interview) using the random route method in the general population aged 14–95; temporal comparison with analogue study conducted in 2018 [16] Beutel ME, Hettich N, Ernst M et al. (2021): OP: 02.05.–29.06.2020 (n=2,503) compared to CP: May–July 2018 (n=2,516)	c	Depressive and anxiety symptoms	PHQ-4 (M)	**Increase** of depressive symptoms to M=1.14; SD=1.23 compared to M=0.89; SD=1.21 in the CP [16] **Increase** of anxiety symptoms to M=1.05; SD=1.31 compared to M=0.77; SD=1.17 in the CP [16]
**Data basis: Regional Study of the Central Institute of Mental Health Mannheim** Survey study (written) in the Mannheim area in the general population aged 18–65; comparison over time with study from 2018 with the same survey design [10] Kuehner C, Schultz K, Gass P et al. (2020): OP: 24.04.–23.05.2020 compared to CP 2018 (n=721)	a	Wellbeing	WHO-5-Well-Being-Index	**Unchanged** in general population to 2018 (2020: M=56.52; 2018: M=56.83) [10]
	b	Psychosocial distress	PHQ-D Stress Module (total)	**Unchanged** in general population compared to 2018 [10]
	c	Symptoms and syndrome diagnosis: Depressive disorder, anxiety disorder, Somato-form disorder Comorbidity syndrome diagnosis.	Screening-based syndrome diagnoses from PHQ-D Sum values of the respective symptoms	**Unchanged** in general population (Mannheim region) in OP versus CP (PHQ-D syndrome diagnosis 2020: 29.4%; 2018: 26.8%; PHQ-D comorbidity diagnosis 2020: 20.3%; 2018: 8.9%) [10]

STUDY TYPE B: Representative cross-sectional studies in trend design with random sampling from an access panel and identical survey design of the temporal comparative data [17–21]				
Study or data basis and publications (including observation period etc.)	Results			
	Outcome		Operationalisation/measuring instrument	Description and interpretation by authors of the publication
**Data basis: COSMO – COVID-19 Snapshot Monitoring** Survey study (online): weekly each approx. 1,000 persons aged 18–74 years since 03.03.2020 with random drawing via a panel provider. A representative distribution of the data by age × gender as well as federal state is aimed for. Education is not taken into account. Comparison over time by presenting the weekly course, partly additional comparison with previous norm data. [21] COSMO – COVID-19 Snapshot Monitoring: Resilience (2021): OP: until 01.06.2021 [20] COSMO – COVID-19 Snapshot Monitoring: Resources and stresses (2021): OP: until 14.07.2021 [19] Gilan D, Röthke N, Blessin M et al. (2020): OP: t1: 24.03.2020, t2: 31.03.2020, t3: 21.04.2020 each compared with norm sample	a	Satisfaction of life – information until 14.07.2021	Single item (Scale 1–7)	**Unchanged** in general population and stable over the course of the pandemic (until 14.07.2021): M=4.6 to M=4.8 [20]
	b	Situational stress – information until 14.07.2021	Single item	High burden in general population at the beginning of the pandemic (52% burdened) – **decline** to 33% in September 2020, since then **increase** to peak of 61% in early February 2021, **decline** since early May 2021 to approximately the level of summer 2020 [20] **Increase** in all age groups since September 2020, especially in the 18–29 age group (69% burdened in January and April 2021); **decrease** since May 2021 [20]
	b	Mental distress (anxiety, dejection) – information until 09.03.2021	Five single items from GAD-7, ADS, IES-R (M)	**Increase** in anxiety in general population at onset of pandemic (24.03.2020: M=0.77; SD=0.94 and 31.03.2020: M=0.75; SD=0.91) compared to norm sample (M=0.50; SD=0.64), **decline** until end of April 2020 (M=0.60; SD=0.83) [19], then level relatively **unchanged** until 09.03.2021 [20] **Increase** in dejection in the general population at the beginning of the pandemic (M=0.66; SD=0.88) compared to the norm sample (M=0.43; SD=0.70), level **unchanged** until April 2020 [19], **increase** in younger people until 09.03.2021 [20] Risk factors for high psychological stress: young age, female gender, having children, single parent, migration background, living alone or more than two persons in household [19]
	a	Resilience – Information until 14.07.2021	Brief-Resilience-Scale (BRS)	**Unchanged** in general population at start of pandemic (M=3.45; SD=0.85) compared to norm sample 2018 (M=3.49; SD=0.84) [19, 21], **decrease** over course of pandemic [21] (24.03.2020: M=3.43 compared to 01.06.2021: M=3.25) Older people more resilient than younger people, older people more resilient during the pandemic also compared to the 2018 norm sample [21]
**Data basis: German-Depression-Barometer** Survey study (online): COVID-19 focus conducted twice since the beginning of the pandemic in general population aged 18–69 years; based on RESPONDI panel; comparison over the time of the two observation periods [18] Stiftung Deutsche Depressionshilfe 2020 (2020): OP: 06/2020 + 07/2020 (n=5,178) [17] Stiftung Deutsche Depressionshilfe 2021 (2021): OP: 02/2021 (n=5,135)	b	Mental distress	Single items	**Increase** in the feeling of low mood in the general population(71% perceive second lockdown as depressing compared to 59% in the first lockdown compared to 36% in summer 2020) [17, 18] **Increase** in severe family stress in the general population (25% in the second lockdown compared to 16% in summer 2020) [17] **Increase** in general population’s perception that fellow citizens are inconsiderate (second lockdown 46% vs. first lockdown 40%) [17]

STUDY TYPE C: Representative, one-off cross-sectional studies with random drawing without an identical survey design of the temporal comparative data [22–25]				
Study or data basis and publications (including observation period etc.)	Results			
	Outcome		Operationalisation/measuring instrument	Description and interpretation by authors of the publication
**Data basis: Corona-COMPASS-Study** Survey study (online) with a random sample from a commercial panel (payback panel, no possibility for participants to self-select); eligible population with online access aged 18–70 (n=8,977, of which 5,844 were interviewed twice), comparison over time with previous SOEP surveys (see study type A) [24] Huebener M, Waights S, Spiess K et al. (2020): OP: 01.05.–01.07.2020 versus CP 2018 (n=8,977)	a	Satisfaction of life	Short scale L-1 (Scale 0–10)	**Decrease** in general population compared to 2018: M=6.95 compared to M=7.36 [24] **Decrease** among parents with children younger than 16 years, women and people with low education; highest life satisfaction among older persons (>65 years) [24]
**Data basis: Parent Study of the Berlin University Alliance** Survey study (telephone, online) of parents of minor children aged 18–73 years, random sample of Germanspeaking households with minor children; comparison over time with previous norm samples and retrospective self-assessment. [23] Calvano C, Engelke L, Di Bella J et al. (2021): Survey from 03.08.11.08.2020; (n=1,024) plus erratum [25]	b	Stress experience	Parental Stress Scale (total) for time of subjectively highest stress (OP) and for January 2020 (CP)	**Increase** in general population (parents of minor children) at time of highest exposure so far during pandemic versus retrospective estimate for January 2020: M=36.93; SD=10.45 compared to pre-COVID-19 M=34.72; SD=10.63 [23]
	c	Depressive and anxiety symptoms	PHQ-4 (M) for time of subjectively highest stress (OP)	**Increase** in general population (parents of minor children) at time of highest stress compared to pre-pandemic norm sample; depressive symptoms: M=1.38 in the OP compared to M=0.94 in the CP; symptoms of anxiety: M=1.14 in the OP compared to M=0.82 in the CP [23, 25]
**Data basis: Study of the elderly population aged 65 and over by the University of Leipzig** Survey study (telephone), random sample of older people aged 65–94 in the general population; comparison over time with previous norm samples. [22] Röhr S, Reininghaus U, Riedel-Heller S G (2020): OP 06.04.–25.04.2020	c	Psychopathological symptoms	Brief Symptom Inventory (BSI-18)	**Unchanged** in general population (persons 65 years and older) compared to pre-pandemic norm levels (M: 1.4 vs. 2.0; 1.6 vs. 1.6; 2.2 vs. 2.4 and 5.1 vs. 6.0) [22]

STUDY TYPE D: Cohort studies with representative initial sample and random sampling at t1 [26, 27]				
Study or data basis and publications (including observation period etc.)	Results			
	Outcome		Operationalisation/measuring instrument	Description and interpretation by authors of the publication
**Data basis: NAKO Health Study** Survey study (online): special longitudinal survey of the NAKO Health Study; baseline survey t1 as random sample from 18 study regions (2014–2019, n=205,219); comparison over time with t2: re-participants at t2 aged 20–74 years (n=113,928, approx. 55% of t1, no difference in re-participation by age, gender, lower in north-eastern study sites). [26] Peters A, Rospleszcz S, Greiser K H et al (2020): OP: 30.04.–29.05.2020 (n=113,928) versus CP (t1: 2014–2019) [27] Berger K, Riedel-Heller S, Pabst A et al. (2021): OP: 30.04.–29.05.2020 (n=113,928) versus CP (t1: 2014–2019)	c	Depressive symptoms	PHQ-9 Cut-Off: >9 Total; mean difference to t1	**Increase** in the general population (in the study sites) to 8.8% (t2) compared to 6.4% (t1) and by an average of 0.3 compared to CP [27]. Increase is only seen in persons under 60 years of age in the mean difference of 0.38+/-0.02 points [26]; clearest increase in youngest age group; stronger in regions with higher incidence and in persons tested positive for COVID-19 [26]
	c	Symptoms of generalised anxiety disorder	GAD-7 Cut-Off: >9 Total; mean difference to t1	**Increase** in the general population (in the study sites) to 5.7% (t2) compared to 4.3% (t1) and 0.45 for women and for men 0.2 [27] Increase is only seen in persons under 60 years of age in the mean difference of 0.38+/-0.02 points [26]; clearest increase in youngest age group; stronger in regions with higher incidence and in persons tested positive for COVID-19 [26]
	b	Psychosocial stress burden	PHQ-D stress module Total; mean difference at t1	**Increase** in the general population (in the study sites) in the mean difference by 0.36+/-0.02 points in all age groups [26] Increase stronger in regions with higher incidence and in individuals who tested positive for COVID-19 [26]

STUDY TYPE E: Longitudinal studies with non-probability initial sample at t1 [28–32]				
Study or data basis and publications (including observation period etc.)	Results			
	Outcome		Operationalisation/measuring instrument	Description and interpretation by authors of the publication
**Data basis: LORA – Longitudinal Resilience Assessment** Survey study (online) as a special survey of the LORA study (=prospective study of healthy adults with a focus on resilience, Rhine-Main region): since 31.03.2020 with the start of the first lockdown in Germany, implementation of eight weekly surveys; participants aged 18–50 years (M=31.5±8.4), 69% female without differences to the LORA initial sample; comparison over the time during the pandemic and compared to the last measurement before the outbreak event [29, 32] Ahrens KF, Neumann RJ, Kollmann B et al. (2021): OP: from 31.03. through eight weekly surveys (t1: n=523, t8: n=451) compared to CP: last measurement before pandemic	b	Everyday burdens per week	Amount	**Decrease** in sample to [32] to 41.2±22.3 in week 8 versus 60.0±27.2 before the pandemic [29]
	c	Mental symptoms	GHQ-28 Difference, M	Average **decrease** [32] in sample to 16.2±7.1 in weeks 5 to 8 versus 20.5±9.7 before the pandemic [29] Identification of three subgroups when analysing intra-individual changes: continuous **increase** in psychological symptoms at around 8%; at 9% initial **increase** and rapid decrease; at 83% improvement or **unchanged** from pre-pandemic levels [29, 32]
**Data basis: Longitudinal study (project to classify the factorial structure of protective factors influencing health at Saarland University)** Survey study (online), sampling from self-recruiting WiSo panel (sampling procedure not documented in publication); participants aged 20–95 years (M=55.0; SD=13.9), 53.6% female; comparison over time between t1 (before pandemic outbreak) and t2. [31] Schäfer S K, Sopp M R, Schanz C G et al. (2020): t1: 17.02.–23.02.2020, t2: 16.03.–22.03.2020; n=1,591	c	Psychopathological symptoms	Mini-Symptom Checklist Difference	**Unchanged** in entire group (i.e. on average without taking individual change into account). Intra-individual **increase** at about 10%, **decrease** at about 8%, **unchanged** for about 82% of the sample from t1 to t2 [31]
**Data basis: Longitudinal study with adults working fulltime** Survey study (mode not stated); sample from representative online panel: full-time working adults aged 18–69; 59.6% female; comparison over time using four survey time points with t1 before pandemic [30] Zacher H, Rudolph C W (2020): OP: t1: 12/2019, t2: 03/2020; t3: 04/2020, t4: 05/2020 (n=979)	a	General satisfaction of life	Single item (Scale 1 to 7)	Intra-individual **decrease** in general satisfaction of life between March and May 2020, no change between December 2019 and March 2020 [30]
	a	Wellbeing	Items of the Positive and Negative Affect Schedule (Short Form)	Intra-individual **decrease** of positive and negative affect between March and May 2020, no change between December 2019 and March 2020 [30]
**Data basis: CORA – Longitudinal Study of the Charité Berlin** Survey study (online), pandemic-initiated with self-selecting baseline sample at t1 in general population and three follow-up surveys; inclusion with participation in at least one follow-up survey (n=2,376 people (age 18–82) were willing to be interviewed again [see 33]) selective sample with 76.7% female; 89.6% higher education or health workers; Temporal comparison over time over the course of the pandemic [28] Bendau A, Plag J, Kunas S et al. (2020): OP: 27.03.–15.06.2020 (t1: 03/2020, t2: 04/2020, t3: 05/2020, t4: 06/ 2020)	c	Depressive and anxiety symptoms	PHQ-4 (Cut-Off >5); M, Median, Difference	**Decrease** in sample from 31.0% (t1) to 22.6% (t4) [28] Continuous decrease in the mean value of the sample across all measurement points; especially decrease in symptom severity; decrease in the median value from 4 (t1) to 3 (t4) [28] Intra-individual **increase** in about 25% of the sample from t1 to t4 [28]
	c	Depressive symptoms	PHQ-2 (Cut-Off >2); M, Median, Difference	**Decrease** in sample from 32.7% (t1) to 25.3% (t4) [28] Continuous decrease in the mean value of the sample across all measurement points; especially decrease in symptom severity; no decrease in the median value [28] Intra-individual **increase** in about 25% of the sample from t1 to t4 [28]
	c	Symptoms of generalised anxiety disorder	GAD-2 (Cut-Off >2); M, Median, Difference	**Decrease** in sample from 36.4% (t1) to 24.9% (t4) [28] Continuous decrease in the mean value of the sample across all measurement points; especially decrease in symptom severity; decrease in the median value from 2 (t1) to 1 (t4) [28] Intra-individual **increase** in about 10% of the sample from t1 to t4 [28]
	b	COVID-19-related anxiety	C-19-A M, Median, Difference	Continuous decrease in the mean value of the sample across all measurement points; **decrease** in the median value from 9 (t1) to 4 (t4) [28] Intra-individual **increase** in about 10% of the sample from t1 to t4 [28]

STUDY TYPE F: Cross-sectional studies with online non-probability sample [33–45]				
Study or data basis and publications (including observation period etc.)	Results			
	Outcome		Operationalisation/measuring instrument	Description and interpretation by authors of the publication
**Data basis: Online survey of the LVR-Klinikum Essen** Cross-sectional survey 1 (10.03.–27.07.2020): Sample composition differs from the general population at all evaluation points (approx: 70% female, 75% with high education (at least Abitur), 61% younger than 44 years). Standardisation by calculation of weighting factors for the distribution of characteristics in the general population is not described. Since the second wave of infection, the study has been continued as a new cross-sectional study. [34] Moradian S, Bäuerle A, Schweda A et al. (2021): OP: 15.03.–04.04.2020 versus CP: 02.11–22.11.2020; adjustment of the two samples has been conducted (n=7,288) [35] Bäuerle A, Steinbach J, Schweda A et al. (2020): OP: 10.03.–05.05.2020; temporal comparison with retrospective assessment of the same instrument before the pandemic (n=15,037) [36] Bäuerle A, Teufel M, Musche, V et al. (2020): OP: 10.03.–05.05.2020 compared to norm sample, adjustment of samples not described (n=15,037) [37] Hetkamp M, Schweda A, Bäuerle A et al. (2020): OP: 10.03.–30.04.2020; comparison over time in the pandemic and against CP: norm sample, adjustment of samples not described (n=16,245) [38] Skoda EM, Spura A, De Bock F et al. (2021): OP: 10.03.–27.07.2020; comparison over time with norm sample before the pandemic and during the pandemic by dividing into five pandemic phases (n=16,918)	c	Depressive symptoms	PHQ-2 (Cut-Off >2), M	14.3% (OP) in sample vs. 7.6% (CP: retrospective assessment of instrument before pandemic); mean difference significant; interpreted as **increase**, especially among persons with pre-existing mental disorder [35] 14.3% (OP) in sample versus 5.6% (CP: norm sample); interpreted as **increase** [36] Lockdown 2 > Lockdown 1 in samples; interpreted as increase [34] **Increase** in sample from 8% in initial phase to 17% during phase 2 and 21% during phase 3 (crisis, lockdown), stable level afterwards; plus **increase** compared to 5.6% in pre-COVID-sample [38]
	c	Symptoms of generalised anxiety disorder	GAD-2 (Cut-Off >2), M	19.7% (OP) in sample vs. 9.0% (CP: retrospective assessment of instrument before pandemic); mean difference significant; interpreted as **increase** [35]
	c	Symptoms of generalised anxiety disorder	GAD-7 (Cut-Off >9, at least moderate symptoms)	16.8% (OP) in sample vs. ~ 6% (CP: norm sample); interpreted as **increase** [36] **Increase** in the sample over the course of the pandemic from 7% to 37% to 22%. Comparison with norm sample interpreted as up to 8-fold increase in prevalence in population exposed to COVID-19 [37] **Increase** in sample from 9% in initial phase to 22% each during phases 2 and 3 (crisis, lockdown), then slight drop to still higher level (22% and 20% respectively) compared to baseline values; plus increase in sample compared to norm sample of ~ 6%, interpreted as 2 to 10-fold increase in fear during pandemic [38] **Unchanged** in samples during the pandemic: lockdown 1=lockdown 2 [34]
	b	Mental distress	Distress Thermometer (Cut-Off >3)	65.2% (OP) in sample vs. 51.8% (CP: retrospective assessment of instrument before the pandemic); mean difference significant; interpreted as **increase** [35] 65.2% (OP) in sample vs. 39% (CP: norm sample); interpreted as **increase** [36] **Unchanged** in samples during the pandemic: lockdown 1=lockdown 2 [34] **Increase** in sample from 51% in initial phase to 63% during phase 2 and 60% during phase 3 (crisis, lockdown), then stable level of 58%; plus increase in sample compared to norm sample with 39% [38]
	b	COVID-19 related anxiety	Single item (Scale 1–7)	**Increase** in sample at the beginning of the pandemic, then **decrease** below baseline level [37, 38]
**Data basis: CORA [see 28] Initial sample – Charité Berlin** Survey study (online; self-selecting) in general population 18 years and older; selective composition: 70.1% female; 82.4% higher education (at least university entrance qualification); comparison over time with norm sample before pandemic, adjustment of samples not described [33] Petzold MB, Bendau A, Plag J et al. (2020): OP: 27.03.–06.04.2020 (n=6,509) versus CP: Publication from 2010	c	Depressive and anxiety symptoms	PHQ-4, M	4.15 in sample vs. 1.76 in norm sample; interpreted as hypothesis-generating indication of possible **increase** [33]
**Data basis: Initial sample of a longitudinal study of the University Tübingen** Survey (online; self-selecting) in general population aged 18–85 years with the aim of identifying protective factors for mental health; selective composition: 76.6% female; 81.4% higher education (at least university entrance qualification); comparison over time with representative pre-pandemic data without sample adjustment. [39] Bauer LL, Seiffer B, Deinhart C et al. (2020): OP: 08.04.–26.04.2020 versus CP: representative data from 2013 (n=3,700)	c	Depressive symptoms	PHQ-9 (>9,. at least moderate msymptoms)	31.4% in OP compared to 8.1% in CP; interpreted as an **increase** in the prevalence of depressive disorders [39]
	c	Symptoms of panic disorder	PHQ-Panic Module Long Version	5.7% in OP vs. 2.0% in CP; interpreted as increase in the prevalence of panic disorders [39]
	c	Symptoms of generalised anxiety disorder	GAD-7 (>9, less. moderate symptoms)	7.4% in OP compared to 2.2% in CP; interpreted as an **increase** in the prevalence of generalised anxiety disorders [39]
**Data basis: Online survey of the Charité Berlin** Survey (online; self-selecting) in general population aged 18–81 years; 75.5% female; temporal comparison over the course of the pandemic (monthly) [40] Liu S, Heinzel S, Haucke MN et al. (2020): OP: April–September 2020 (n=1,903)	b	COVID-19 related stress	COVID-19 Peritraumatic Distress Index (CPDI)	**Increase** of reported psychological distress in the sample over the survey period. Women, young people and lonely people were particularly affected [40]
**Data basis: Online survey of the Medizinische Hochschule Hannover** Survey study (online; self-selecting) in general population aged: M=40.4; SD=11.7; 83% female (separate reporting for women and men); years of education: M=15.9; SD=4.2), comparison over time with norm samples, among others, adjustment of the sample not described [41] Jung S, Kneer J, Krüger T (2020): OP: 01.04.–15.04.2020 (n=3,545)	b	Psychosocial stress burden	PHQ Stress-Module	Women 6.40 and men: 6.19 in sample – men and women are in the range of mild psychosocial stress (Defined range: 5–9) [41]
	c	Depressive and anxiety symptoms	PHQ-4; (M)	Women in the sample 3.91 vs. 1.71 in reference sample; men in the sample: 3.21 vs. 1.31 in reference sample; interpreted as mental distress [41]
	a	Wellbeing	WHO-5 (M)	Women 51.44 vs. 72.6 in reference sample (or: vs. healthy individuals who have an average score of 75); men: 47.52 vs. 68.28 in reference sample (or: vs. healthy individuals who have an average score of 75); interpreted as psychological distress [41]
**Data basis: Initial sample of a longitudinal study of the Justus Liebig University Giessen** Survey study (online; self-selecting) in general population aged 18 years and older; selective composition: 79.5% female; 62.8% students; comparison over time with prevalences of mental disorders based on psychodiagnostic interview and representative data, adjustment of sample not described [42] Munk AJL, Schmidt NM, Alexander N (2020): OP: 27.03.–03.04.2020 (n=949)	c	Symptoms ‘Any mental disorder’	All instruments listed here	50.6% vs. 27.9% 12-month prevalence in general population; interpreted as **increase** [42]
	c	Depressive symptoms	Beck-Depression-Inventory	35.3% vs. 7.7% 12-month prevalence in general population; interpreted as **increase** [42]
	c	Symptoms of generalised anxiety disorder	GAD-7	12.0% vs. 2.2% 12-month prevalence in general population; interpreted as **increase** [42]
	c	Symptoms of panic disorder	PHQ-Panic module	5.4% vs. 2.0% 12-month prevalence in general population; interpreted as **increase** [42]
	c	Symptoms of obsessive compulsive disorder	Obsessive-Compulsive Inventory-R	21.4% vs. 3.6% 12-month prevalence in general population; interpreted as **increase** [42]
**Data basis: Online survey of the universities Marburg and Gießen** Survey study (online; self-selecting) in general population aged 18–81 years; 74.8% female; comparison over time using retrospective assessment of the same measure before the first lockdown [43] Schwinger M, Trautner M, Kärchner H et al. (2020): OP: 01.04–28.05.2020 (n=1,086)	a	General satisfaction of life	Four Items of the Temporal Satisfaction with Life Scale M	2.65 in sample on OP vs. 3.04 retrospective data; interpreted as strong **decrease** [43]
	b	Anxiousness, depressivity	Three items each of the State-Trait-Anxiety-Depression-Inventory (STADI) M	Depressivity 1.91 in sample on OP vs. 1.71 retrospective data; interpreted as moderate **increase** [43] Anxiousness 2.02 in sample on OP vs. 1.79 retrospective data; interpreted as moderate **increase** [43]
**Data basis: Online survey of the universities Göttingen and Regensburg** Survey study (online; self-selecting) in general population aged 18–88 years; (n=1,744, 72.2% female); comparison over time with reference sample from 2010, sample adjustment by random deletion of female participants (n=1,001, 52.2% female) [45] Schelhorn I, Ecker A, Bereznai J et al. (2020): OP: 08.04.–01.06.2020	c	Depressive symptoms	ICD-10-Symptom-Rating (ISR) (> Cut-Offs for moderate or severe symptoms)	13% moderate and 5% severe symptoms in sample vs. 4.8% and 1.1% in reference sample; interpreted as an **indication** of **possible increase**[45] Particularly in women and in young people, the symptoms are more severe [45]
**Data basis: Online survey of the Universities Witten/Herdecke, …** Survey study (online, participant recruitment via snowball system); comparison over time by dividing the sample into three cohorts over the survey period [44] Büssing A, Rodrigues Recchia D, Dienberg T et al. (2021): OP: June 2020 (n=1,333), July–September 2020 (n=823), October–January 2021 (n=625)	a	Wellbeing	WHO-5-Well-being Index (Total)	**Decrease** in the OP October–January 2021 compared to OP June 2020 and OP July–October 2020 [44]
	a	Satisfaction of life	Brief Multidimensional Life Satisfaction Scale	**Decrease** in the OP October–January 2021 compared to OP June 2020 and OP July–October 2020 [44]

**Table:** Category II: Routine Data Abbreviations used: AU = Incapacity for work; EBM = Uniform value scale for billing statutory health insurance services, IRR = Incidence Rate Ratio; PEPP = fixed rate remuneration system in psychiatry and psychosomatics (PEPP); AMDP = Working Group for Methodology and Documentation in Psychiatry; Information on Coded Diagnoses (FXX.X) according to the International Statistical Classification of Diseases and Related Health Problems (ICD-10)

ROUTINE DATA [46–69]				
Study or data basis and publications (including observation period etc.)	Results			
	Outcome		Operationalisation/measuring instrument	Description and interpretation by authors of the publication
**Data basis: Outpatient care data from the National Association of Statutory Health Insurance Physicians** Data from persons with statutory health insurance who use SHI-accredited medical care; first to second quarter 2020 data from 16 of the 17 SHI-accredited medical associations (excluding Bremen), third to fourth quarter 2020 from 15 (excluding Bremen and Mecklenburg-Western Pomerania). [68] Mangiapane S, Zhu L, Czihal T et al. (2020): 14 defined observation periods in 2020: t0: 01.01.–03.03.; t1: 04.03.–10.03.; t2: 11.03.–17.03.; t3: 18.03.–24.03.; t4: 25.03.–31.03.; t5: 01.04.–28.04.; t6: 29.04.–26.05.; t7: 27.05.–30.06.; t8: 01.07.–28.07.; t9: 29.07.–25.08.; t10: 26.08.–30.09.; t11: 01.10.–04.11.; t12: 05.11.–02.12.; t13: 03.12.–31.12. compared to the corresponding comparison periods in 2019	d	Utilisation of outpatient care by medical and psychological psychotherapists and specialists in psychosomatic medicine and psychotherapy	Number of treatment cases	**Decrease** compared to the same period of the previous year in the periods t2 to t6 with the strongest decrease at t4 with 36.5% and 39.8%, one-time increase at t7 by 24.4% and 17.8%;at t8-t11 **fluctuations** around the level of the previous year, t12–t13 **decrease** by 14.9% and 12.4% compared to the same period of 2019; Changes over time roughly comparable to other doctor groups with the exception of the comparatively strong increase at t7 [68]
	d	Utilisation of outpatient psychotherapy	Number of treatment cases for psychotherapeutic services for individual therapy (section 35.2.1 EBM) and group therapy (section 35.2.2 EBM)	Individual therapy: **decrease** compared to previous year’s period from t2 to t6 with strongest decrease at t3 by 23.3%, then **increase** compared to previous year’s periods until t11 with strongest increase at t11 (3.8%), then **decrease** again in t13 by 3,0% [68] Group therapies **decrease** compared to previous year’s periods from t2 to t8 with strongest decrease at t5 by 59.8%, in t9 to t11 roughly **unchanged** at previous year’s level, **decrease** again at t12 and t13 by up to 16.4% [68]
	d	Utilisation of outpatient substitution treatment	Number of treatment cases for substitution treatment for drug dependence (section 1.8 EBM)	**Decrease** compared to previous year’s period from t3 to t8 with strongest decrease at t4 by 13.0%, from t9 to t13 fluctuating below previous year’s level with decrease between 1.1% and 3.2% [68]
**Data basis: Outpatient care data from the Disease Analyzer-Database (IQVIA)** Data from people with use of medical practices that are part of the panel of the Disease Analyzer Database (IQVIA) [54] Jacob L, Smith L, Koyanagi A et al. (2020): 03–06/2020 vs. 03–06/2019 (n=1,140 GP practices, n=1,854,742 people with treatment cases in 01–06/2020). [55] Michalowsky B, Hoffmann W, Bohlken J et al. (2020): 02–05/2020 compared to 02–05/2019 (n=1,095 GP or internal medicine practices and n=960 specialist practices, of which n=155 neurological and psychiatric practices; n=2,447,356 persons aged ≥65 years with treatment cases)	d	Outpatient diagnostic incidence and prevalence of anxiety disorders in GP practices	Number of persons with (first) diagnosis of F41.0–3 or F41.8–9	**Increase** in diagnoses by 19% in 2020 compared to 2019, increase in first diagnoses by 21% [54]
	d	Outpatient diagnostic incidence of anxiety disorders in GP practices by age	Number of persons with first diagnosis of F41.0–3 or F41.8–9 by age	**Decrease** in the proportion of 18–30 year olds to 16.8% in 2020 compared to 20.3% in 2019 [54]
	d	Comorbidity of outpatient incident diagnoses in general practitioners of anxiety disorders	Number of persons with first diagnosis of F41.0–3 or F41.8–9 and diagnosis of nine somatic disorders	**Increase** in people with comorbid COPD in 2020 to 9.4% compared to 7.9% in 2019 and with comorbid asthma to 11.3% in 2020 compared to 9.7% in 2019 [54]
	d	Utilisation of outpatient care by psychiatric/neurological practices among persons aged ≥65 years	Number of physician contacts in psychiatric/neurological practices	Increase of 13% in March, **decrease** of 18% in April and 12% in May compared to 2019; rather small decrease compared to 7 other doctor groups with a mean of 5% (rank 3) [55]
	d	Referral or admission to outpatient or inpatient care by psychiatric/neurological practices among persons aged ≥65 years	Number of referrals or admissions in outpatient or inpatient care by psychiatric/neurological practices	**Decrease** of 22% in referrals and 40% in admissions in March to May 2020 compared to 2019 [55]
	d	Outpatient diagnosis of depression for persons aged ≥65 years	Number of Persons with first diagnosis F32–33	**Decrease** of 13% in 2020 compared to 2019 [55]
**Data basis: Work incapacity data for KKH** Data of those compulsorily and voluntarily insured with the KKH (excluding the unemployed and pensioners), n=812,000 [57] [56] KKH Kaufmännische Krankenkasse (2020): 01–06/2020 compared to 01–06/2019 [58] KKH Kaufmännische Krankenkasse (2021): 2020 compared to 2019 [57] KKH Kaufmännische Krankenkasse (2021): 2020 compared to 2019 and 2019 compared to 2018	d	Incapacity to work cases	Number of cases with incapacity to work due to F-diagnosis	**Increase** of approx. 80% compared to 2019 [56]
	d	Duration of incapacity to work by gender	Number of days per case of incapacity to work due to F-diagnosis	**Increase** for women by ‘just under 4 days’, for men by ‘almost five days’ in 2020 compared to 2019 [58]
	d	Duration of incapacity to work	Number of days per case of incapacity to work due to F-diagnosis	**Increase** of 4.2 days in 2020 compared to 2019, larger increase than from 2018 to 2019 of 3.9 days [57]
**Data basis: Incapacity to work for BARMER** Data from working people aged 15–64 who are insured with BARMER [49] BARMER (2020): 01–06/2020 compared to 2019	d	Number of work days lost due to sickness	Proportion of days with incapacity to work due to F-diagnosis in all insured days	**Decrease** of 5.5% in the period April–June 2020 compared to April–June 2019 [49]
**Data basis: Incapacity to work data for BKK** Data from employees insured with participating BKKs [51] BBK (2021): 01/2020 –04/2021	d	Number of work days lost due to sickness	Proportion of days with incapacity to work due to F-diagnosis in all insured days	**Decrease** from March (0.83%) to May 2020 (0.70%), renewed **increase** as of November 2020 (0.78%) and February/March 2021 (0.80%) [51]
**Data basis: Incapacity to work data for DAK** Data from employed persons insured with the DAK [52] DAK Gesundheit (2021): 01–12/2020 compared to 01–12/2019	d	Incapacity to work cases	Number of cases of incapacity to work due to F-diagnosis per 100 insured persons	**Decrease** in 2020 compared to 2019 for women from 9.3 to 8.8 and for men from 5.7 to 5.1 [52]
	d	Duration of incapacity to work	Number of days per case of incapacity to work due to F-diagnosis	**Decrease** in short case durations (1–7 days) by 20% in 2020 compared to 2019, increase in long case durations (>6 weeks) by 6% in 2020 compared to 2019 [52]
	d	Work incapacity cases according to diagnosis	Number of days of incapacity to work due to F-diagnosis per 100 insured persons according to individual diagnoses	Changes in 2020 vs. 2019: **decrease** in somatoform disorder (F45) by 6%, almost **unchanged** in depression (F32+33) with an increase of 0.3%; **increase** in other anxiety disorders (F41) by 5%, reactions to severe stress and adjustment disorders (F43) by 8% and other neurotic disorders (F48) by 7% [52]
**Data basis: Incapacity to work data for AOK** Data from employees insured with the AOK, n=approx. 13 million [47] Wissenschaftliches Institut der AOK (WIdO) (2020): 01.01.–31.08.2020 compared to 01.01.–31.08.2019	d	Incapacity to work cases	Number of cases of incapacity to work due to F-diagnosis per 100 insured persons	**Decrease** to 11.1 in the period January to August 2020 from 12.0 in the comparison period in 2019 [47]
	d	Duration of incapacity to work	Number of days per case of incapacity to work due to F-diagnosis	**Increase** to 29.3 in the period January to August 2020 from 25.9 days in the same period in 2019 [47]
**Data basis: Work incapacity data for TK** Data from persons insured with TK who are subject to social insurance or registered as unemployed, n=5.4 million [67] Techniker Krankenkasse (2021): 2020 compared to 2019 [66] Techniker Krankenkasse (2021): 01–12/2020 com-pared to 2019/ 2018	d	Days of incapacity to work	Number of days of incapacity to work due to F-diagnosis per 100 years insured	**Increase** of 5.1 days for men and 14.4 days for women in 2020 compared to 2019 [67]
	d	Change in sick leave from 2019 to 2020 compared to 2018 to 2019	Change in number of days with incapacity to work due to F-diagnosis	**Increase** of 0.11 days from 2019 to 2020 (2.98) compared to increase from 2018 to 2019 (2.89) [66]
**Data basis: Emergency admission of the psychiatric clinic of the Hannover Medical School** Data of persons aged ≥18 years who presented at the psychiatric emergency admission of Hannover Medical School and had medical contact with psychiatrists [62] Seifert J, Meissner C, Birkenstock A et al. (2021): 16.03.–24.05.2020 compared to 16.03.–24.05.2019, n=374 presentings	d	Psychiatric emergencies according to clinical characteristics	Number of cases with F-diagnosis, after repeated presentations within one month and suicide attempts before presentation	**Decrease** in case numbers of 21.4% in the period 16.03.–24.05.2020 compared to the same period in 2019, at the same time **increase** in the number of repeated presentations within one month to 30.2% compared to 20.4% in 2019; **unchanged** proportion of suicide attempts [62]
	d	Psychiatric emergencies according to diagnoses	Number of diagnoses of mental disorders in the main diagnostic groups F1–F4 and F6, as well as ‘other’ (F0, F5, and F7–9)	**Decrease** to 15.2% in the period 16.03–24.05.2020 from 22.2% in the same period in 2019 for affective disorders (F32–33), at the same time **increase** for personality and behavioural disorders (F6) to 12.3% from 7.8%, **unchanged** proportion for F1, F2, F4 and ‘other’ [62]
	d	Aspects of the psycho-pathological findings	Documentation of 65 selected symptoms of psychopathological findings according to AMDP system	**Increase** in the period 16.03.–24.05.2020 compared to the same period in 2019 for formal thinking disorder (75.7% compared to 65.1%), hopelessness (13.9% compared to 5.3%) and social withdrawal (14.4% compared to 8.0%), all others **unchanged** (including suicidality) [62]
	d	Relation between psychiatric emergencies and the COVID-19-pandemic	Explicit documentation of the association of the case with the COVID-19-pandemic by the treating psychiatrist	A relation between the presentation and the COVID-19-pandemic was documented for 20.1% of all persons; among these, the proportion of persons with suicide attempts **increased** 3-fold compared to cases without a connection to the COVID-19-pandemic [62]
**Data basis: Emergency admission of the Central Institute of Mental Health Mannheim** Data of persons that presented in the emergency admission of the Central Institute of Mental Health Mannheim [61] Hoyer C, Ebert A, Szabo K et al. (2020): 01.01.–19.04.2020 compared to 01.01.–21.04.2019	d	Presentations in psychiatric emergency admission	Number of presentations in psychiatric emergency admission	**Decrease** of 26.6% in presentation rates 18.03.–14.04./01.01.–17.03 in 2020 compared to 2019 [61]
	d	Presentations in psychiatric emergency admission according to diagnosis	Number of presentations in psychiatric emergency admission by main diagnosis group	**Decrease** of 42.3% in showcase rates 18.03.–14.04./01.01.–17.03 in 2020 compared to 2019 for affective disorders [61]
**Data basis: Emergency admission and consultation requirements of the Klinikum rechts der Isar, Technische Universität München** Data from people who presented at the central interdisciplinary emergency admission of the Klinikum rechts der Isar, Technische Universität München [60] Aly L, Sondergeld R, Holzle P et al. (2020): 21.03.–01.05.2020 vs 23.03.–03.05.2019 (n=3,549 total number of all presentations in emergency admission)	d	Psychiatric emergencies	Number of cases with corresponding coding according to Manchester-Triage-System or ICD-10	**Unchanged** absolute number of psychiatric emergencies as well as distribution among ICD main diagnosis groups, but **increase** in the share of all presentations to 5% compared to 3% in the previous year [60]
	d	Psychiatric consultation requirements, connection with the COVID-19-pandemic	Proportion of cases with psychiatric consultation requests with content related to the COVID-19-pandemic and with suicide attempts	A substantive link to the COVID-19-pandemic was found in 21%; among these, an **increased** proportion of suicide attempts (22%) was observed compared to those without a COVID-19 link (6%) [60]
**Data basis: Emergency admission and inpatient care in HELIOS-Clinics** Persons admitted as psychiatric emergencies to one of n=67 hospitals of the HELIOS Group with main diagnosis F00–F69 [59] Fasshauer JM, Bollmann A, Hohenstein S et al. (2021): 13.03.–21.05.2020 vs. 01.01.–12.03.2020; 13.03.–21.05.2019 (n=13,151 total number of all psychiatric emergency admissions)	d	Psychiatric Emergencies	Number of cases with main diagnosis F00–F69	**Decrease** in 13.03.–21.05.2020 compared to the previous months in 2020 (IRR 0.68) and the comparative period in 2019 (IRR 0.70), affected all main diagnosis groups [59]
	d	Length of stay for psychiatric diagnosis	Number of days of inpatient treatment for cases with main diagnosis F00–F69	**Decrease** in length of stay to 9.8 in 13.03.–21.05.2020 compared to 14.7 in the previous months in 2020 and 16.4 in the comparative period in 2019, respectively [59]
**Data basis: AOK inpatient care data** Data of inpatient treatment among insured persons of the AOK, n=27 million [46] Wissenschaftliches Institut der AOK (WIdO) (2020): 16.03.–05.04.2020 compared to 16.03.2019–05.04.2019 [48] Wissenschaftliches Institut der AOK (WIdO) (2021): 01/2020–02/2021 compared to 01–12/2019	d	Inpatient treatment cases	Number of inpatient treatment cases for F-diagnoses	**Decrease** of 49% in 16.03.–05.04.2020 compared to the same period in 2019, thus comparatively strong decrease (rank 6) among the 21 treatment events considered [46]
	d	Inpatient cases in psychiatric, psychotherapeutic and psychosomatic clinics and departments	Number of treatment cases in specialised hospitals or departments for psychiatry and psychotherapy, child and adolescent psychiatry and psychotherapy and psychosomatic medicine and psychotherapy that bill according to the PEPP system	**Decrease** compared to 2019 in the entire observation period, most pronounced in April 2020 by 31% as well as in January 2021 by 21%, least pronounced in September 2020 by 5% [48]
**Data basis: LVR clinics inpatient care data** Data of persons who were admitted to one of n=9 psychiatric clinics of the Landschaftsverband Rheinland (LVR) as day-clinic patients or inpatients [50] Zielasek J, Vrinssen J, Gouzoulis-Mayfrank E (2021): 18.03.–31.05.2020 compared to 18.03.–31.05.2019 (n=10,545 treatment cases)		Day-clinic and inpatient admissions per day	Number of admitted cases to day-clinic and inpatient care (planned admissions and emergencies) per day in clinics for psychiatry, child and adolescent psychiatry, psychosomatic medicine, geriatrics and addiction treatment	**Decrease** of 25% in the period 18.03.–31.05.2020 compared to the same period in 2019 [50]
		Day-clinic patient and inpatient cases by diagnosis	Discharge diagnosis according to main diagnosis groups	**Decrease** in the period 18.03.–31.05.2020 compared to the same period in 2019 for intelligence disorders (F7) with 51%, neurotic, stress and somato-form disorders (F4) with 35%, affective disorders (F3) with 34% and personality and behavioural disorders (F6) with 31%, organic mental disorders including Alzheimer’s (F0/G3) with 10% and schizophrenia, schizotypal and delusional disorders (F2) with 9% [50]
**Data basis: Statistics on narcotics-related deaths from the Land Offices of Criminal Investigation** [53] The Federal Government Drug Commissioner (2021): 2020 compared to 2019	d	Deaths due to narcotics use	Total number of deaths in all federal states	**Increase** of 13% to 1,581 in 2020 compared to 1,398 in 2019 [53]
**Data basis: Telephone counselling ‘Telefonseelsorge’** Data from persons who made contact with the nation-wide telephone counselling ‘Telefonseelsorge’ in one of n=91 counselling centres [64] Arendt F, Markiewitz A, Mestas M et al. (2020): 01.01.–31.03.2020 compared to 01.01.–31.03.2019 (contact via telephone) [65] Armbruster S, Klotzbücher V (2020): 01.01.2019–28.04.2020 (contact via telephone or online)	d	Calls to telephone counselling	Number of calls to telephone counselling	**Increase** to approx. 700 calls more per day at the end of March 2020 compared to the same period in 2019 [64]
	d	Contacts to telephone counselling	Number of contacts to telephone counselling per day (telephone and online)	**Increase** of approx. 20% around 22.03.2020, after approx. three weeks of **decline**, in April still increased compared to previous year’s level; stronger increase in federal states with stricter infection control measures [65]
	d	Reasons for contacting telephone counselling	Central counselling topics for each contact to telephone counselling	**Increase** in counselling topics ‘mental and physical health’, ‘relationship/social issues’ and ‘violence’ [65]
**Data basis: Cause of death statistics of the city of Leipzig** [63] Radeloff D, Papsdorf R, Uhlig K et al. (2020): 16.03.–24.05.2020 compared to 16.03.–24.05.2019 and previous years 2010–2019; 3 phases depending on the limitation of social contacts and mobility: No restriction (01.01.–16.03.2020), moderate restriction (17.–21.03., 06.06.–30.09.2020), severe restriction (22.03.–05.06.2020) compared to previous years as of 2010	d	Suicide rate	Number of suicides per 100,000 life years	**Decrease** in suicide rate from 16.8 (time without restrictions) to 7.2 (time of severe restraints) due to unexpectedly high suicide rates in 2019 and early 2020; **unchanged** values in comparison none vs. mild and mild vs. severe restraints; no differences between expected and observed trends based on previous year trends [63]
**Data basis: Cause of death statistics of the Federal Statistical Office** [69] Federal Statistical Office (2021): 2020 vs. 2019	d	Suicide	Total number of suicides (cause of death X60–X84)	**Decrease** in suicides to n=8,565 in 2020 compared to n=9,041 in 2019 [69]

## Appendix

**Annex Table 4:** Indicators Category I: Primary data

OUTCOME TYPE A: INDICATORS OF POSITIVE MENTAL HEALTH				
Study type	Indicator	Inventory	Data source/Study	Publication
**Construct: Satisfaction of life**				
A	Satisfaction of life	Short scale L-1 (Scale 0–10)	SOEP	[1–7]
B	Satisfaction of life	Single item (Scale 1–7)	COSMO	[8]
C	Satisfaction of life	Short scale L-1 (Scale 0–10)	Corona COMPASS	[9]
E	Satisfaction of life	Single item (1 to 7)	Longitudinal study of people in full-time employment	[10]
F	Satisfaction of life	Four items of the Temporal Satisfaction with Life Scale	Online survey of the universities Marburg and Giessen	[11]
F	Satisfaction of life	Brief Multidimensional Life Satisfaction Scale	Online survey of the universities Witten/Herdecke, …	[12]
**Construct: Wellbeing**				
A	Wellbeing	Four single items (Scale 1–5): Positive and negative affect	SOEP	[1, 2, 4–7]
A	Wellbeing	WHO-5-Well-Being-Index	Study of the Central Institute of Mental Health Mannheim	[13]
E	Wellbeing	Items of the Positive and Negative Affect Schedule (Short Form)	Longitudinal study of people in full-time employment	[10]
F	Wellbeing	WHO-5	Online survey of the Medizinische Hochschule Hannover	[14]
F	Wellbeing	WHO-5-Well-Being-Index	Online survey of the universities Witten/Herdecke, …	[12]
**Construct: Resilience**				
B	Resilience	Brief-Resilience-Scale (BRS)	COSMO	[15, 16]
**3 Constructs**			**8 Data sources/Studies**	**[1–16]**

OUTCOME TYPE B: INDICATORS OF MENTAL BURDEN				
Study type	Indicator	Inventory	Study	Publication
**Construct: Feelings of anxiety, dejection**				
A	Anxiety	State-Trait Anxiety Inventory short scale until week 4: Five items (M; cut-off >2) from week 5: Two items	The Mannheim Corona Study	[17–19]
B	Mental distress (anxiety, dejection)	Five single items from GAD-7, ADS, IES-R (M)	COSMO	[8, 15]
F	Anxiety, depression	State-Trait-Anxiety-Depression-Inventory (STADI)	Online survey of the universities Marburg and Giessen	[11]
**Construct: COVID-19-related anxiety and distress**				
E	COVID-19 specific anxiety	C-19-A	CORA Longitudinal study	[20]
F	COVID-19 related anxiety	Single item (Scale 1–7)	Online survey of the LVR-Clinic Essen	[21, 22]
F	COVID-19 related stress	COVID-19 Peritraumatic Distress Index (CPDI)	Online survey by Charité	[23]
**Construct: Situational burden and psychosocial stress**				
A	Psychosocial distress	PHQ-D Stress Module (Total)	Study of the Central Institute of Mental Health Mannheim	[13]
B	Situational stress	Single item	COSMO	[8]
B	Mental distress	Single item	German Depression Barometer	[24, 25]
C	Stress experience	Parental Stress Scale (Total)	Parent study of the Berlin University Alliance	[26, 27] Erratum
D	Psychosocial distress	PHQ-D stress module (Total); mean difference at t1	NAKO	[28]
E	Everyday burdens per week	Amount	LORA	[29, 30]
F	Psychosocial distress	PHQ-D Stress Module	Online survey of the Medizinische Hochschule Hannover	[14]
F	Mental distress	Distress Thermometer (Cut-Off >3)	Online survey of the LVR-Clinic Essen	[22, 31–33]
**3 Constructs**			**13 Data sources/Studies**	

OUTCOME TYPE C: INDICATORS OF ACUTE SYMPTOMS OF MENTAL DISORDER				
Study type	Indicator	Inventory	Study	Publication
**General psychopathological symptoms**				
A	Psychopathological symptoms	Brief Symptom Inventory (BSI-18)	Study of Ulm University	[34]
A	Symptoms and syndrome diagnosis: depressive disorder, anxiety disorder, somatic disorder; psychosocial distress comorbidity syndrome diagnosis	Screening-based syndrome diagnoses from PHQ-D total values of the respective symptomatology	Study of the Central Institute of Mental Health Mannheim	[13]
C	Psychopathological symptoms	Brief Symptom Inventory (BSI-18)	Study of the elderly population aged 65 and over by the University Leipzig	[35]
E	Mental symptoms	GHQ-28 median value	LORA	[29, 30]
E	Psychopathological symptoms	Mini-Symptom Checklist	Longitudinal study of Saarland University	[36]
F	Symptoms ‘Any mental disorder’	Beck-Depression-Inventory, GAD-7, PHQ-Panic Module, Obsessive-Compulsive Inventory-Revised	Initial sample of a longitudinal study of the Justus Liebig University Giessen	[37]
**Depressive and anxiety symptoms**				
A	Depressive and anxiety symptoms	PHQ-4 (M)	SOEP	[1, 2, 4, 6, 7, 38]
A	Depressive and anxiety symptoms	PHQ-4 (M)	Study of the universities Mainz and Leipzig	[39]
C	Depressive and anxiety symptoms	PHQ-4 (M)	Parent study of the Berlin University Alliance	[26, 27] Erratum
E	Depressive and anxiety symptoms	PHQ-4 (Cut-Off >5); M	CORA Longitudinal study	[20]
F	Depressive and anxiety symptoms	PHQ-4; (M)	Online survey of the Medizinische Hochschule Hannover	[14]
F	Depressive and anxiety symptoms	PHQ-4; (M)	CORA initial sample in the cross-section	[40]
**Depressive symptoms**				
A	Depressive symptoms	PHQ-8 (>9)	GEDA-EHIS	[41, 42]
A	Depressive symptoms	Individual symptoms PHQ-8 over time	GEDA-EHIS	[41, 42]
A	Depressive symptoms	from week 5 PHQ-2	The Mannheim Corona Study	[18]
D	Depressive symptoms	PHQ-9 (Cut-Off: >9 total; median difference)	NAKO	[28, 43]
E	Depressive symptoms	PHQ-2 (Cut-Off >2)	CORA Longitudinal study	[20]
F	Depressive symptoms	PHQ-2 (Cut-Off >2)	Online survey of the LVR-Clinic Essen	[22, 31–33]
F	Depressive symptoms	PHQ-9 (>9, at least moderate Symptomatology)	Initial sample of a longitudinal study of the University Tübingen	[44]
F	Depressive symptoms	Beck-Depression-Inventory	Initial sample of a longitudinal study of the Justus Liebig University Giessen	[37]
F	Depressive symptoms	ICD-10-Symptom-Rating (ISR)	Online survey of the universities Göttingen and Regensburg	[45]
**Symptoms of anxiety disorder**				
D	Symptoms of generalised anxiety disorder	GAD-7 (Cut-Off: >9, total; median difference to t1)	NAKO	[28, 43]
E	Symptoms of generalised anxiety disorder	GAD-2 (Cut-Off >2)	CORA Longitudinal study	[20]
F	Symptoms of generalised anxiety disorder	GAD-7 (Cut-Off >9, at least moderate Symptomatology)	Online survey of the LVR-Clinic Essen	[21, 22, 32, 33]
F	Symptoms of generalised anxiety disorder	GAD-2 (Cut-Off >2)	Online survey of the LVR-Clinic Essen	[31]
F	Symptoms of panic disorder	PHQ Panic Module Long Version	Initial sample of a longitudinal study of the University Tübingen	[44]
F	Symptoms of generalised anxiety disorder	GAD-7 (>9, at least moderate symptoms)	Initial sample of a longitudinal study of the University Tübingen	[44]
F	Symptoms of generalised anxiety disorder	GAD-7	Initial sample of a longitudinal study of the Justus Liebig University Giessen	[37]
F	Symptoms of panic disorder	PHQ-Panic module	Initial sample of a longitudinal study of the Justus Liebig University Giessen	[37]
**Symptoms of obsessive compulsive disorder**				
F	Symptoms of obsessive compulsive disorder	Obsessive-Compulsive Inventory-Revised	Initial sample of a longitudinal study of the Justus Liebig University Giessen	[37]
**5 Constructs**			**18 Data sources/Studies**	

**Category II: Routine data**

**Table:**

INDICATORS OF CARE PATTERNS AND MORTALITY OF MENTAL DISORDERS			
Indicator	Operationalisation	Data body or holder	Publication
**Area of care: Crisis service**			
Calls to telephone counselling	Number of calls to telephone counselling	Telephone counselling (nationwide)	[46]
Contacts to telephone counselling	Number of contacts to telephone counselling per day (telephone and online)	Telephone counselling (nationwide)	[47]
Reasons for contacting telephone counselling	Central counselling topics for each contact to telephone counselling	Telephone counselling (nationwide)	[47]
**Area of care: Outpatient care**			
Utilisation of outpatient care by medical and psychological psychotherapists and specialists in psychosomatic medicine and psychotherapy	Number of treatment cases	Contractual physician billing data from the Central Research Institute of Ambulatory Health Care in Germany	[48]
Utilisation of outpatient psychotherapy	Number of treatment cases for psychotherapeutic services for individual therapy (section 35.2.1 EBM) and group therapy (section 35.2.2. EBM)	Contractual physician billing data from the Central Research Institute of Ambulatory Health Care in Germany	[48]
Utilisation of outpatient substitution treatment	Number of treatment cases for substitution treatment for drug dependence (section 1.8 EBM)	Contractual physician billing data from the Central Research Institute of Ambulatory Health Care in Germany	[48]
Outpatient diagnostic incidence and prevalence of anxiety disorders in GP practices	Number of persons with (first) diagnosis of F41.0–3 or F41.8–9	Physician billing data from the practice panel of the Disease Analyser Database (IQVIA)	[49]
Outpatient diagnostic incidence of anxiety disorders in GP practices by age	Number of persons with first diagnosis of F41.0–3 or F41.8–9 by age	Physician billing data from the practice panel of the Disease Analyser Database (IQVIA)	[49]
Comorbidity of outpatient incident diagnoses in general practitioners of anxiety disorders	Number of persons with first diagnosis of F41.0–3 or F41.8–9 and diagnosis of 9 somatic disorders	Physician billing data from the practice panel of the Disease Analyser Database (IQVIA)	[49]
Utilisation of outpatient care by psychiatric/neurological practices among persons aged ≥65 years	Number of physician contacts in psychiatric/neurological practices	Physician billing data from the practice panel of the Disease Analyser Database (IQVIA)	[50]
Referral or admission to outpatient or inpatient care by psychiatric/neurological practices among persons aged ≥65 years	Number of referrals or admissions in outpatient or inpatient care by psychiatric/neurological practices	Physician billing data from the practice panel of the Disease Analyser Database (IQVIA)	[50]
Outpatient diagnosis of depression for persons aged ≥65 years	Number of persons with first diagnosis F32–33	Physician billing data from the practice panel of the Disease Analyser Database (IQVIA)	[50]
**Area of care: Incapacity to work**			
Incapacity to work cases	Number of cases with incapacity to work due to F-diagnosis	Data about incapacity to work from KKH	[51]
Duration of incapacity to work by gender	Number of days per case of incapacity to work due to F-diagnosis	Data about incapacity to work from KKH	[52]
Duration of incapacity to work	Number of days per case of incapacity to work due to F-diagnosis	Data about incapacity to work from KKH	[53]
Number of work days lost due to sickness	Proportion of days per case of incapacity to work due to F-diagnosis	Data about incapacity to work from BARMER	[54]
Number of work days lost due to sickness	Proportion of days per case of incapacity to work due to F-diagnosis	Data about incapacity to work from BKK	[55]
Incapacity to work cases	Number of cases of incapacity to work due to F-diagnosis 100 per insured persons	Data about incapacity to work from DAK	[56]
Duration of incapacity to work	Number of days per case of incapacity to work due to F-diagnosis	Data about incapacity to work from DAK	[56]
Work incapacity cases according to diagnosis	Number of days of incapacity to work due to F-diagnosis per 100 insured persons according to individual diagnoses	Data about incapacity to work from DAK	[56]
Incapacity to work cases	Number of cases of incapacity to work due to F-diagnosis per 100 insured persons	Data about incapacity to work from AOK	[57]
Duration of incapacity to work	Number of days per case of incapacity to work due to F-diagnosis	Data about incapacity to work from AOK	[57]
Days of incapacity to work	Number of days of incapacity to work due to F-diagnosis per 100 years of insured persons	Data about incapacity to work from TK	[58]
Change in sick leave from 2019 to 2020 compared to 2018 to 2019	Change in number of days with incapacity to work due to F-diagnosis	Data about incapacity to work from TK	[59]
**Area of care: Stationary care**			
Psychiatric emergencies according to clinical characteristics	Number of cases with F-diagnosis, after repeated presentations within one month and suicide attempts before presentation	Routine data from the emergency department of the psychiatric clinic of the Medizinische Hochschule Hannover	[60]
Psychiatric emergencies according to diagnoses	Number of diagnoses of mental disorders in the main diagnostic groups F1–F4 and F6, as well as ‘other’ (F0, F5, and F7–9)	Routine data from the emergency department of the psychiatric clinic of the Medizinische Hochschule Hannover	[60]
Aspects of the psychopathological findings	Documentation of 65 selected symptoms of psychopathological findings according to AMDP system	Routine data from the emergency department of the psychiatric clinic of the Medizinische Hochschule Hannover	[60]
Linking the psychiatric emergency to the COVID-19 pandemic	Explicit documentation of the association of the case with the COVID-19 pandemic by the treating psychiatrist	Routine data from the emergency department of the psychiatric clinic of the Medizinische Hochschule Hannover	[60]
Presentations in psychiatric emergency admission	Number of presentations in psychiatric emergency admission	Routine data of emergency admission of the Central Institute of Mental Health Mannheim	[61]
Presentations in psychiatric emergency admission after diagnosis	Number of presentations in psychiatric emergency admission by main diagnosis group	Routine data of emergency admission of the Central Institute of Mental Health Mannheim	[61]
Psychiatric emergencies	Number of cases with corresponding coding according to Manchester Triage System or ICD-10	Routine data of emergency admission and inpatient care of the Klinikum rechts der Isar, Technische Universität München	[62]
Psychiatric consultation requirements, connection with the COVID-19 pandemic	Proportion of cases with psychiatric consultation requests with content related to the COVID-19 pandemic and with suicide attempts	Routine data of emergency admission and inpatient care of the Klinikum rechts der Isar, Technische Universität München	[62]
Psychiatric emergencies	Number of cases with main diagnosis F00–F69	Routine data of emergency admission and inpatient care in HELIOS-Clinic	[63]
Length of stay for psychiatric diagnosis	Number of days of inpatient treatment for cases with main diagnosis F00–F69	Routine data of emergency admission and inpatient care in HELIOS-Clinic	[63]
Inpatient treatment cases	Number of inpatient treatment cases for F-diagnoses	Data about incapacity to work from AOK	[64]
Inpatient cases in psychiatric, psychotherapeutic and psychosomatic clinics and departments	Number of treatment cases in specialised hospitals or departments for psychiatry and psychotherapy, child and adolescent psychiatry and psychotherapy, and psychosomatic medicine and psychotherapy that bill according to the PEPP system	Data about incapacity to work from AOK	[65]
Day-clinic and inpatient admissions per day	Number of admitted cases to day-clinic and inpatient care (planned admissions and emergencies) per day in clinics for psychiatry, child and adolescent psychiatry, psychosomatic medicine, geriatrics and addiction treatment	LVR clinics inpatient care data	[66]
Day-clinic and inpatient cases by diagnosis	Discharge diagnosis according to main diagnosis groups	LVR clinics inpatient care data	[66]
**Subject area: Mortality**			
Deaths due to narcotics use	Total number of deaths in all federal states	Statistics on narcotics-related deaths from the Land Offices of Criminal Investigation	[67]
Suicide rate	Number of suicides per 100,000 life years	Cause of death statistics of the city of Leipzig	[68]
Suicide	Total number of suicides (cause of death ICD X60-X84)	Cause of death statistics (nationwide) of the Federal Statistical Office	[69]
**5 Care or subject areas**		**18 Data sources**	**[46-69]**
