# Corrigendum: Mental health of the adult population in Germany during the COVID-19 pandemic. Rapid Review

**DOI:** 10.25646/13256

**Published:** 2025-07-11

**Authors:** Elvira Mauz, Sophie Eicher, Diana Peitz, Stephan Junker, Heike Hölling, Julia Thom

Mauz E, Eicher S, Peitz D et al. (2021)

Journal of Health Monitoring 6(S7): 2–63.

DOI 10.25646/9537

Corrigendum, page 2:

For the first author, the following affiliation was added: Charité-Universitätsmedizin, Institute of Medical Sociology and Rehabilitation Science, Berlin, Germany.

The updated version of the article is available at DOI 10.25646/9537.2

